# Mouse SLX4 Is a Tumor Suppressor that Stimulates the Activity of the Nuclease XPF-ERCC1 in DNA Crosslink Repair

**DOI:** 10.1016/j.molcel.2014.03.014

**Published:** 2014-05-08

**Authors:** Michael R.G. Hodskinson, Jan Silhan, Gerry P. Crossan, Juan I. Garaycoechea, Shivam Mukherjee, Christopher M. Johnson, Orlando D. Schärer, Ketan J. Patel

**Affiliations:** 1 MRC Laboratory of Molecular Biology, Francis Crick Avenue, Cambridge, CB2 0QH, UK; 2 Department of Chemistry, Stony Brook University, Stony Brook, NY 11794-3400, USA; 3 Department of Pharmacological Sciences, Stony Brook University, Stony Brook, NY 11794-8651, USA; 4 Department of Medicine, Level 5, Addenbrooke’s Hospital, University of Cambridge, Cambridge CB2 0QQ, UK

## Abstract

SLX4 binds to three nucleases (XPF-ERCC1, MUS81-EME1, and SLX1), and its deficiency leads to genomic instability, sensitivity to DNA crosslinking agents, and Fanconi anemia. However, it is not understood how SLX4 and its associated nucleases act in DNA crosslink repair. Here, we uncover consequences of mouse *Slx4* deficiency and reveal its function in DNA crosslink repair. *Slx4*-deficient mice develop epithelial cancers and have a contracted hematopoietic stem cell pool. The N-terminal domain of SLX4 (mini-SLX4) that only binds to XPF-ERCC1 is sufficient to confer resistance to DNA crosslinking agents. Recombinant mini-SLX4 enhances XPF-ERCC1 nuclease activity up to 100-fold, directing specificity toward DNA forks. Mini-SLX4-XPF-ERCC1 also vigorously stimulates dual incisions around a DNA crosslink embedded in a synthetic replication fork, an essential step in the repair of this lesion. These observations define vertebrate SLX4 as a tumor suppressor, which activates XPF-ERCC1 nuclease specificity in DNA crosslink repair.

## Highlights

• SLX4 suppresses tumorigenesis and contributes to the maintenance of HSPC pool in mice
• SLX4 enhances the nuclease activity of XPF-ERCC1 up to 100-fold
• In complex, SLX4-XPF-ERCC1 efficiently unhooks a DNA crosslink

## Graphical Abstract

**Figure:**
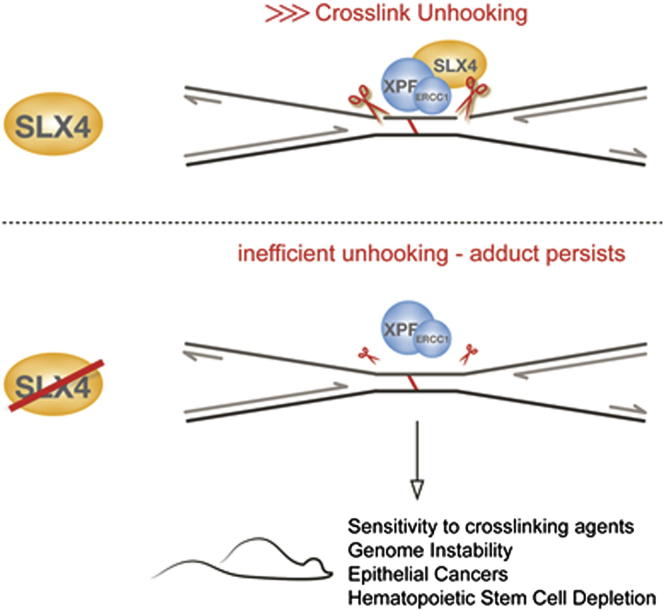

## Teaser

SLX4 interacts with multiple nucleases that can facilitate the repair of DNA interstrand crosslinks (ICLs). Hodskinson et al. assess the long-term consequences of Slx4 deficiency in mice and show that the interaction between SLX4 and XPF-ERCC1 nuclease can support ICL repair. Biochemical analysis reveals that SLX4 stimulates XPF-ERCC1 nuclease to cut replication intermediates and unhook an ICL.

## Introduction

Organisms have evolved mechanisms to preserve genome integrity, without which their DNA is prone to the accumulation of damage and mutation (Lindahl, 1993). DNA damage occurs from exposure to exogenous mutagens and endogenous reactive processes, including DNA replication (Barnes and Lindahl, 2004). Because there are many sources of damage, the chemical nature of modified DNA can be very diverse, necessitating specific mechanisms of DNA damage recognition and repair. Interstrand crosslinks (ICLs) are a particularly deleterious form of DNA damage. If they are not removed, ICLs block DNA replication, whereas their incomplete repair can also lead to the accumulation of double-strand breaks (DSBs) (Auerbach and Wolman, 1976). Although we do not know how such lesions naturally arise, they are readily formed when cells are exposed to chemotherapeutic agents, such as cisplatin. As an ICL covalently joins opposite strands of DNA together, its resolution is a complex process requiring multiple steps.

The best insight into the mechanism of ICL repair has been gained from elegant studies using *Xenopus* egg extracts in vitro (Knipscheer et al., 2009; Long et al., 2011; Räschle et al., 2008). This system enabled the replication-coupled repair of a single site-specific ICL to be followed at nucleotide resolution. In this system, two replication forks converge upon the crosslink, pausing 20 nucleotides (nt) away from the lesion. Subsequently, one fork progresses, and when it reaches the lesion, dual incision at either side of the ICL occurs. These cleavage events occur on the opposite, lagging strand template from advancing replication (Räschle et al., 2008). This critical step is known as “unhooking” and allows sister chromatid separation. The lesion is then bypassed using a trans-lesion synthesis (TLS) polymerase, with subsequent extension being facilitated by the Rev1-Rev7-Rev3 complex (Knipscheer et al., 2009; Niedzwiedz et al., 2004; Räschle et al., 2008; Simpson and Sale, 2003). This results in the regeneration of an intact sister chromatid that can serve as a template for homologous recombination (HR) to repair the residual DSB (Long et al., 2011). The identity of the proteins responsible for this complex repair process have largely been identified by genetic means in several organisms and can be classified into four major groups: (1) the Fanconi anemia proteins (the genes encoding these are mutated in the human chromosome breakage illness Fanconi anemia [FA]), (2) structure-specific endonucleases required to unhook the crosslink, (3) DNA polymerases that bypass lesions, and (4) DNA double-strand break repair proteins that function in HR.

Three of these groups have obvious roles in DNA repair, but until recently the exact function of the Fanconi proteins in ICL repair remained elusive. The primary function of the upstream components of the Fanconi DNA repair pathway is to monoubiquitylate two proteins: FANCD2 and FANCI (Garcia-Higuera et al., 2001; Smogorzewska et al., 2007). Upon monoubiquitylation, these two key repair factors are recruited to chromatin. Monoubiquitylated FANCD2 is required to promote incisions at the site of the lesion: its depletion results in a failure to unhook a crosslink (Knipscheer et al., 2009). Despite this major advance in understanding, the identity of the nuclease(s) that unhook crosslinked DNA remains unclear—deficiency in any one of six nucleases (XPF-ERCC1, MUS81-EME1, SLX1, SNM1A, SNM1B, or FAN1) leads to cellular hypersensitivity to DNA crosslinking agents (Castor et al., 2013; Demuth et al., 2004; Kratz et al., 2010; Liu et al., 2010; MacKay et al., 2010; Sengerová et al., 2012; Smogorzewska et al., 2010; Wang et al., 2011; Wyatt et al., 2013).

Although all six nucleases are required to protect cells from the toxic effects of crosslinking agents, it is unlikely that they all act in the same pathway as FA proteins. SNM1A and SNM1B are members of a conserved exonuclease family, so their involvement in ICL repair probably follows the primary incisions at a crosslink (Wang et al., 2011). FAN1 can physically interact with FANCD2, though the physiological relevance of this interaction is unclear, because humans with FAN1 deficiency do not develop FA (MacKay et al., 2010; Trujillo et al., 2012; Zhou et al., 2012). Additionally, genetic analysis has revealed that *FAN1* does not act in a common pathway with the Fanconi genes to repair an ICL (Yoshikiyo et al., 2010). MUS81-EME1-deficient cells are hypersensitive to crosslinking agents, but MUS81-EME1 knockout mice are fertile, in stark contrast to all FA knockout mice (Dendouga et al., 2005; McPherson et al., 2004). An *Slx1*-deficient mouse also shares similarities to *Mus81*-deficiency, again suggesting a nonoverlapping function with the FA genes (Castor et al., 2013). However, as no human patients lacking MUS81-EME1 or SLX1 have yet been described, it is still possible that these nucleases might constitute extremely rare FA complementation groups.

In contrast, mutations in the nuclease XPF-ERCC1 can lead to FA, albeit with more severe clinical features than most common complementation groups (Bogliolo et al., 2013; Kashiyama et al., 2013). Furthermore, *Ercc1-*deficient mice also recapitulate many aspects of FA, such as bone marrow dysfunction, sterility, and developmental defects (Hsia et al., 2003; McWhir et al., 1993; Prasher et al., 2005). However, this key nuclease has additional roles in other repair processes, including gene conversion, single strand annealing, and nucleotide excision repair (NER) (Al-Minawi et al., 2008; Niedernhofer et al., 2001; Sijbers et al., 1996). Despite this, it has been shown that XPF-ERCC1 is required for efficient crosslink unhooking in vivo (Bhagwat et al., 2009; De Silva et al., 2000). In addition, in-vitro-purified XPF-ERCC1 is able to unhook a crosslink, though it does so with poor efficiency (Fisher et al., 2008; Kuraoka et al., 2000).

Taken together, the genetic and biochemical evidence implicate XPF-ERCC1 as the most likely nuclease to unhook a DNA crosslink in the context of the FA pathway. But does XPF-ERCC1 achieve this on its own? Intriguingly, XPF-ERCC1, MUS81-EME1, and SLX1 all interact with a large protein—SLX4—that is thought to act as a scaffold (Andersen et al., 2009; Fekairi et al., 2009; Muñoz et al., 2009; Svendsen et al., 2009). Surprisingly, *SLX4* deficiency in humans leads to classical Fanconi anemia, and *Slx4*-deficient mice phenocopy many aspects of this human illness (Crossan et al., 2011; Kim et al., 2011; Stoepker et al., 2011). Cells lacking *Slx4* are hypersensitive to DNA crosslinking agents, and this defect can only be complemented by SLX4 polypeptides that retain the interaction with XPF-ERCC1 (Crossan et al., 2011; Kim et al., 2013). Furthermore, sequential deletion and mutation analysis revealed that defects in the interaction with MUS81-EME1 and SLX1 are marginally responsible for the function of SLX4 in DNA crosslink repair (Castor et al., 2013; Kim et al., 2013). Thus, an SLX4-XPF-ERCC1 complex could be the key incision nuclease that unhooks DNA crosslinks in vertebrates.

In this study, we assess the long-term consequences of *Slx4* deficiency in mice. We define a minimal SLX4 polypeptide that only interacts with XPF-ERCC1 nuclease that can support crosslink repair. Biochemical analysis of this mini-SLX4-XPF-ERCC1 complex reveals that SLX4 vigorously stimulates XPF-ERCC1 nuclease to cut replication intermediates and unhook an ICL.

## Results

### Mouse *Slx4* Deficiency Leads to Epithelial Cancer Predisposition and Reduced Blood Stem Cells

**Figure 1:**
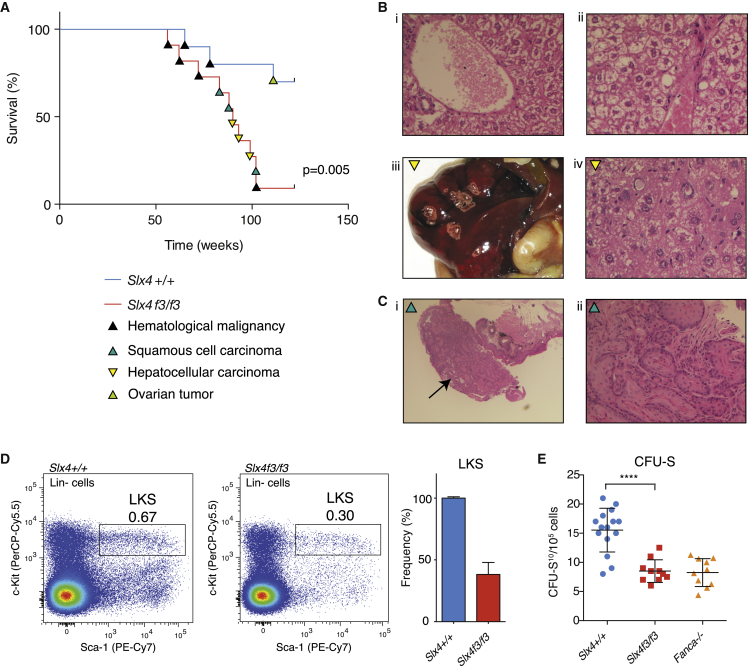
*Slx4*-Deficient Mice Are Cancer Prone and Have a Compromised HSPC Pool (A) Kaplan-Meier curve showing the tumor-free survival of our cohort of aged *Slx4*^*f3/f3*^ C57BL/6NTac mice (n = 28) and congenic controls (n = 28). (B) Hematoxylin and eosin staining of sections of liver in (1) 8-week-old and (2) 24-week-old *Slx4*^*f3/f3*^ mice, revealing karyomegaly and steatosis. (3) Gross pathology of a typical hepatic mass in *Slx4*^*f3/f3*^. (4) Histology of *Slx4*^*f3/f3*^ hepatic mass, showing a primary hepatocellular cancer. (C) (1) Low-power magnification of an anal mass (black arrow), and (2) higher magnification shows features of a typical squamous cell carcinoma with keratin whorls of the rectum. (D) Flow cytometry analysis of total bone marrow from *Slx4*^+/+^ and *Slx4*^*f3/f3*^ mice stained with hematopoietic stem and progenitor cell markers (Linage^−^c-kit^+^Sca1^+^: LKS box). (E) Spleen colony forming assay (CFU-S_10_) was performed in lethally irradiated recipients revealing a reduction in the *Slx4*^*f3/f3*^ bone marrow. Error bars represent SEM. ^∗∗∗∗^p < 0.0001.

We previously characterized homozygous mice carrying the *Btbd12*^tm1a(EUCOMM)Wtsi^ allele (hereafter referred to as *Slx4*^*f3*^). These mice have been maintained for many generations in a pure C57BL/6NTac background. Homozygous *Slx4*^*f3/f3*^ mice were born at sub-Mendelian ratios, were sterile, prone to developmental defects, and hematological cytopenias—these features persist in our colony following transmission of the allele through several generations. Transformed murine embryonic fibroblasts (MEFs) made from these mice were hypersensitive to DNA crosslinks and accumulated broken chromosomes (Crossan et al., 2011). These features bear striking resemblance to FA. We have now followed a cohort of *Slx4*^*f3/f3*^ homozygous mice for up to 2 years: most of these animals succumbed to malignancies within this time frame. The pattern of tumors was atypical, with epithelial-type cancers predominating (rectal squamous cell carcinoma and hepatocellular carcinoma) (Figures 1A–1C). Though some human FA patients develop a range of cancers, most of them have hematopoietic stem cell defects, leading to bone marrow failure (Ceccaldi et al., 2012; Garaycoechea and Patel, 2014). Our previous work showed that the blood from a small proportion of homozygous *Slx4*^*f3/f3*^ mice displayed reduced white blood cell and platelet numbers, prompting us to determine the frequency of hematopoietic stem and progenitor cells (HSPC) residing in the bone marrow of 8- to 12-week old mice. Flow cytometry analysis of the bone marrow for the Lineage^−^c-kit^+^Sca1^+^ (LKS) population shows that this is contracted in *Slx4*^*f3/f3*^ compared to controls (Figure 1D). Furthermore, we carried out a spleen colony forming assay in lethally irradiated recipients, using wild-type or *Slx4*^*f3/f3*^ bone marrow (CFU-S_10_). These data confirm the reduction in the frequency of HSPCs observed by flow cytometry (Figure 1E). In summary, mouse *Slx4* is a tumor suppressor that also functions to preserve hematopoiesis.

### Genetic Analysis and Purification of a Minimal SLX4-XPF-ERCC1 Protein Complex

**Figure 2:**
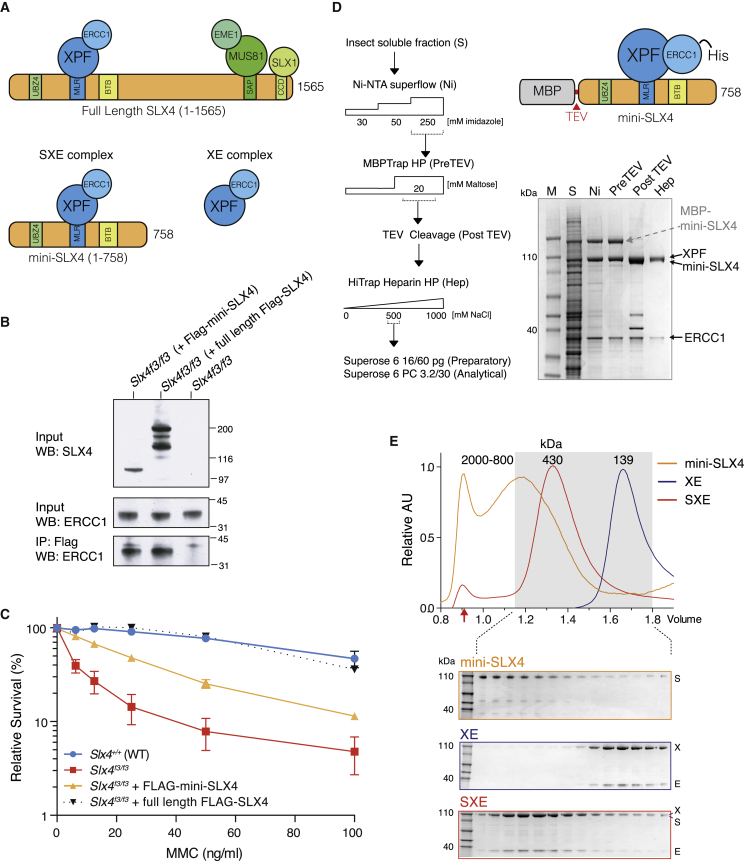
SLX4 1-758 Partially Complements Crosslinker Sensitivity and Can Be Purified in a Complex with XPF-ERCC1 (A) Cartoon depicts the SLX4 polypeptide (1–1565), domains, and interactions with the three nucleases: XPF-ERCC1, MUS81-EME1, and SLX1. A truncated SLX4 1-758 (mini-SLX4) contains the region that interacts with XPF-ERCC1. (B) Full-length FLAG-tagged SLX4 or FLAG-tagged mini-SLX4 was expressed in *Slx4*^*f3/f3*^ MEFs. Anti-FLAG immunoprecipitation shows that the ectopically expressed SLX4 polypeptides can be copurified with XPF-ERCC1. Note: ectopically expressed full-length SLX4 is prone to degradation/aggregation, accounting for the three bands seen by western blot (WB). (C) MTS viability of *Slx4*^*f3/f3*^ MEFs stably expressing full-length FLAG-SLX4 or FLAG-mini-SLX4, exposed to varying doses of Mitomycin C (MMC) for 4 days. (D) Expression and purification of a recombinant mini-SLX4 (1–758) in complex with XPF-ERCC1 (SXE) from insect cells. The purification scheme is shown next to a Coomassie gel depicting the various stages of purification. (E) Analytical gel filtration and Coomassie gels of purified mini-*S*LX4, *X*PF-*E*RCC1 (XE), and SXE complexes. Italicized letters correspond to the proteins shown on the Commassie gel. The shaded box represents those fractions loaded on SDS gels (below). A red arrow denotes the column void volume (∼2 MDa). Error bars represent SEM.

As already mentioned, transformed MEF cells lines obtained from *Slx4*^*f3/f3*^ embryos were hypersensitive to ICL agents, such as Mitomycin C (MMC). The introduction of a full-length *Slx4* transgene into these cells can complement this key phenotypic feature. This simple, cell-intrinsic DNA repair defect provides a system for functional dissection of the SLX4 polypeptide. SLX4 is a large 1565 amino acid polypeptide that serves as a binding platform for three nucleases (Figure 2A). An N-terminal MLR domain mediates the interaction with XPF-ERCC1, whereas MUS81-EME1 and SLX1 bind through regions mapping near the C terminus of SLX4 (Fekairi et al., 2009; Kim et al., 2013; Svendsen et al., 2009). Additionally, SLX4 possess two N-terminal UBZ domains and a central BTB/POZ protein dimerization/interaction domain. We created a truncation of mouse SLX4 (SLX4 1-758: mini-SLX4) that includes the XPF-ERCC1 binding region (MLR) and ectopically expressed this in *Slx4*^*f3/f3*^-deficient MEFs. Mini-SLX4 binds to endogenous XPF-ERCC1 as efficiently as the full-length SLX4 polypeptide (Figure 2B). This mini-SLX4 also significantly complements resistance to MMC (Figure 2C) (LD_50_ values: *Slx4*^*f3/f3*^ 4 ng/ml, Mini-SLX4 23 ng/ml, and full-length SLX4 80 ng/ml).

We next studied the biochemical properties of mini-SLX4, to ask if it modulates the function of XPF-ERCC1. Using insect cells we expressed and purified the mini-*S*LX4-*X*PF-*E*RCC1 (SXE) complex, the *X*PF-*E*RCC1 nuclease (XE), or mini-SLX4 alone (Figure 2D; Figure S1 available online). To compare the properties of these proteins, we performed analytical gel filtration and found that mini-SLX4 was polydispersed, forming high-molecular-mass aggregates (Figure 2E). In contrast, SXE formed a stable, monodispersed complex, indicated by a single peak on gel filtration (Figure 2E). We confirmed these observations using light-scattering analysis (Figure S1F). The molecular mass of the SXE complex was 430 kDa, consistent with the formation of homodimeric SXE complex. These results show that mini-SLX4, which only interacts with XPF-ERCC1, was proficient in ICL repair and that recombinant mini-SLX4-XPF-ERCC1 can be readily purified.

### Mini-SLX4 Alters the Nuclease Activity of XPF-ERCC1 on DNA Structures

**Figure 3:**
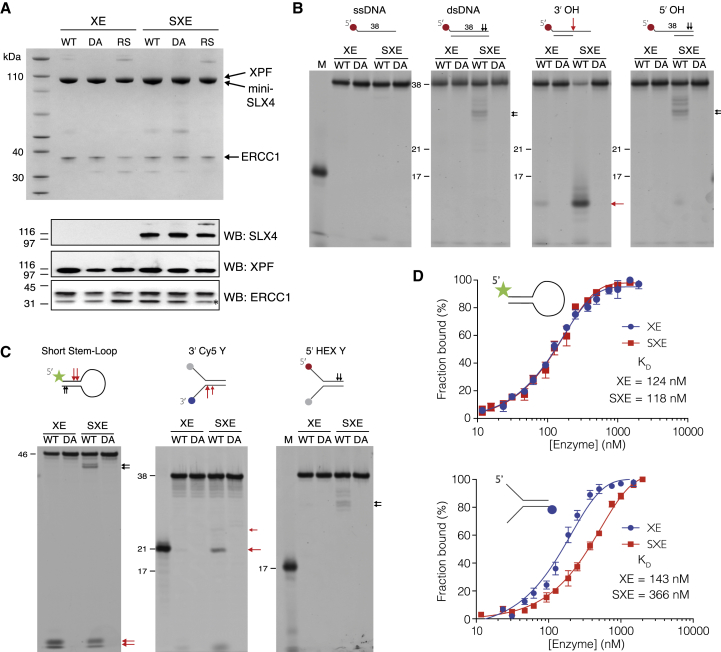
Comparison of the Activities of XE and SXE and Nuclease-Dead Mutants on Various Synthetic DNA Substrates (A) Coomassie gel (top) and western blot (WB) analysis (bottom) of the purified SXE, XE complexes with WT XPF, or catalytically dead mutant D688A (DA) used in the following assays and XPF Fanconi mutation R690S (RS). ERCC1 forms a doublet on WB, owing to a proteolytic site in the N terminus, shifting its mass by ∼2 kDa (^∗^). (B) Activity of WT SXE/XE or DA SXE/XE on DNA structures; single-stranded (ssDNA), double-stranded (dsDNA), 3′ overhang (3′ OH), and 5′ overhang (5′ OH). SXE shows enhanced activity toward 3′ overhangs (red arrow) and also low double-strand nicking activity (black arrow). The colored symbols denote fluorophore-labeled nucleotides. Red arrow marks structure-specific activity. (C) Activity of WT SXE/XE or DA SXE/XE on more complex splayed arms (5′ HEX Y, 3′ Cy5 Y) and stem-loop structures. SXE shows an induction in cleaving the 3′ end of Y-shaped substrate, cleaving near the ss/ds junction (21 nt marker). (D) Fluorescence anisotropy assay to determine binding of SXE and XE to either short stem-loop or splayed arms. Mini-SLX4 does not enhance the binding of XE to either short stem-loop or splayed arms. Normalized and averaged anisotropy ± SEM.

Having established a robust purification strategy for the SXE complex, we set out to compare its nuclease activity with that of XE alone. A critical control was to purify both complexes carrying an XPF point mutation (D688A), known to ablate nuclease activity (Figure 3A; SXE DA and XE DA). The equivalent mutation in human XPF disrupts metal binding at the active site (Figures S2A and S2B) (Enzlin and Schärer, 2002).

A range of DNA oligonucleotides of varying complexity were designed to test the effect of SLX4 on XPF-ERCC1 nuclease activity. These ranged from simple single-stranded DNA (ssDNA) to more complex replication fork and stem-loop substrates (Figure S3A). On ssDNA we observed no activity with the wild-type (WT) complexes, consistent with the structure-specific nature of XPF-ERCC1. SXE showed a weak activity in nicking double-stranded DNA that was not observed for XE alone. Similarly, a 5′ overhang was cut weakly at its duplex end by SXE. This weak activity was always observed in substrates containing a free duplex. SXE showed marked activity when it was presented with a 3′ overhang (Figure 3B). The significant difference came when we tested a short stem-loop structure and Y-shaped substrates, mimicking stalled replication fork (Figure 3C). This stem-loop substrate has frequently been used as a surrogate for NER substrates (Bowles et al., 2012; Enzlin and Schärer, 2002). Both SXE and XE cleaved this short stem-loop structure with similar efficiency (except for the aforementioned weak SXE activity on the duplex end). Surprisingly, we observed a suppression of XE activity on this stem-loop, when we titrated in free mini-SLX4, suggesting the complex requires preassembly for in vitro activity (data not shown). These data are similar to those described in the accompanying study by Klein Douwel et al. (2014), in which full-length *Xenopus* SLX4 exerted a slight inhibitory effect on XPF-ERCC1 activity. In contrast, SXE showed enhanced activity at Y fork (3′ Cy5 Y) structured DNA compared to XE, which produced very little product. Furthermore, this enhancement of structure-specific activity was restricted to the 3′ arm (3′ Cy5 Y) and could not be detected on the 5′ arm (5′ HEX Y) (Figure 3C). The cleavage site was close to the single-strand/double-strand junction (two nucleotides into the duplex), the canonical site for XE cleavage (de Laat et al., 1998). Importantly, the XE or SXE protein complexes, harboring XPF D688A (or D690S, which is described later; data not shown) had no discernable enzyme activity toward any DNA substrate tested.

Finally, we wanted to know if the enhanced activity of SXE on Y-shaped structures was due to enhanced substrate binding. We therefore compared the binding of XE and SXE to both Y and short stem-loop substrates, using fluorescence anisotropy in the absence of metal ions (Figure 3D). Binding to the stem-loop DNA was equivalent for both complexes (K_D_ 124 ± 8 nM and 118 ± 5 nM for XE and SXE, respectively) (Su et al., 2012). When we assayed the Y substrate, surprisingly we found SXE binding was approximately 2-fold lower than XE (K_D_ 143 ± 5 nM and 366 ± 22 nM for XE and SXE, respectively). Cumulatively, the above data suggest that purified SXE is a more potent nuclease than XE; yet, the effect of mini-SLX4 on XPF-ERCC1 activity is more pronounced on specific substrates. Furthermore, this difference is not entirely due to a change in DNA binding, suggesting that SLX4 directly alters the catalytic properties of XPF-ERCC1.

### The SXE Nuclease Complex Is Most Active on Fork-Structured DNA

**Figure 4:**
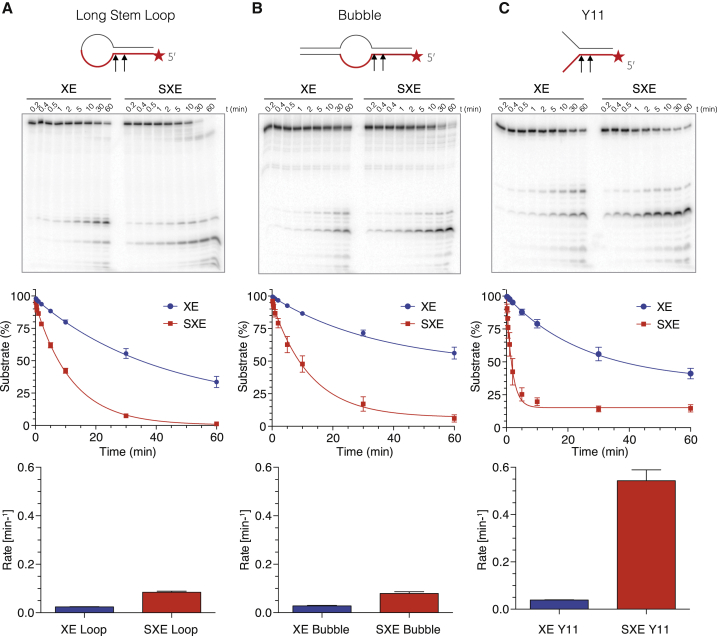
Mini-SLX4 Specifically Enhances XPF-ERCC1 Activity toward Y-Structured DNA (A–C) XE and SXE (5 nM) were reacted with different radiolabeled DNA substrates (∼1.5 pM), over a time course (A, long stem-loop; B, bubble; C, fork-structured DNA [Y11]). Substrates had identical primary sequence around the ss/ds bifurcation (depicted in red). The reaction products were separated by 12% denaturing PAGE gel (top panel), and the decay of the substrate band (S) was quantified and expressed as a percentage of initial substrate (middle panel). Data were fitted using single exponential decay in order to calculate reaction rates (bottom panel). XE data are plotted in blue; SXE data are plotted in red. SXE shows a modest stimulation of activity compared to XE toward stem-loop and bubble substrates and a pronounced induction of activity toward forked DNA (Y11). Error bars represent SEM.

Our qualitative analysis had revealed an effect of mini-SLX4 on XPF-ERCC1 activity toward specific DNA structures. We next sought to test this definitively, assessing the reaction kinetics with an excess of enzyme and divalent metal (Mg^2+^) over substrate. We designed three substrates of identical length and sequence at the ss/ds junction (Figures 4 and S3B). The first two substrates (long stem loop and bubble) were similar to those used to characterize nuclease biochemistry in NER pathways (Enzlin and Schärer, 2002; Evans et al., 1997). Comparison of XE and SXE activities toward this stem loop revealed a modest rate enhancement of SXE (3.7-fold) (Figure 4A). These data differ from those for the short stem-loop substrate previously described, in which we observed marginally less activity of the SXE complex (Figures 3C and S4). The length of the duplex (and a possible contribution of DNA sequence; Bowles et al., 2012) could explain this difference. Next, we assessed the activity of the nuclease complexes toward bubble NER-like substrates (Figure 4B). There was a striking concordance for rates of catalysis of the bubble substrate with data for the stem loop. SXE displayed a similarly modest 3.5-fold induction in catalytic rate compared to XE. However, when we assayed fork-structured DNA, we observed a greater difference between the enzyme complexes (Figure 4C). XE processed the fork substrate (Y11) with similar efficiency to the loop and bubble substrates (half-life 16 min), indicating that in the absence of SLX4 it exhibited very little structural preference (on these substrates). In comparison, the Y11 fork was processed rapidly by SXE (half-life 1 min) compared to XE, a 16-fold increase in catalytic activity. Reaction rates are listed in Table S1. Thus, the structural DNA motif recognized by XE and SXE differs substantially; SLX4 effectively biases XPF-ERCC1 toward processing forked DNA structures.

### SXE Can Unhook a DNA Interstrand Crosslink in Fork-Structured DNA

**Figure 5:**
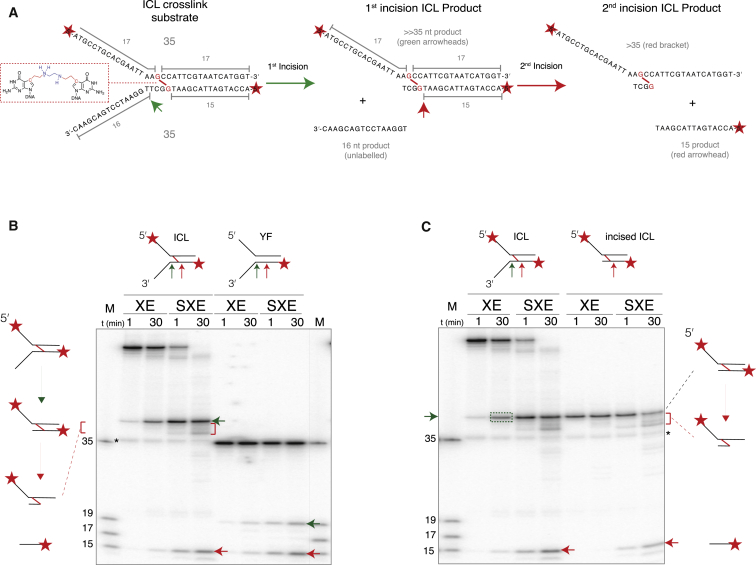
SLX4 Promotes Unhooking of an ICL by XPF-ERCC1 (A) Outline of forked substrate containing a single nitrogen mustard-like interstrand crosslink (ICL) and its predicted reaction products. The substrate was generated from two oligonucleotides (each 35 nucleotides in length) with a crosslink between adjacent guanines, close to the ss/ds junction (red boxed inset). Sequential unhooking of the crosslinked DNA should result in an intermediate product (≫35 nt), followed by final products >35 and ≤15 nt (illustrated with green and red arrowheads). (B) The forked ICL substrate or an identical, but noncrosslinked, control (YF) were radiolabeled at the 5′ end and reacted with XE or SXE enzyme complexes and analyzed by denaturing PAGE. Cleavage sites and reaction products corresponding to those illustrated in (A) are shown as arrows and brackets (the equivalent products from YF migrate at 19 and 15 nt). Comparison of ICL and YF digestion reveals the first ≫35 nt product is most likely to result from an incision at the ss/ds boundary (corresponding to 19 nt product of noncrosslinked YF fork). This was confirmed with 3′-end labeling (Figure S5). (C) The primary reaction product (≫35 nt, green box) from the ICL substrate was purified as a substrate in a second reaction (“incised ICL”) to test whether the ICL was cleaved again (unhooked). The 15 and >35 nt product (red arrowhead and brackets) correspond to cleavage 5′ of the adducted guanine. All reactions contained 5 nM enzyme complex and ∼1.5 pM substrate. An asterisk denotes a low abundance, background band (a contaminant noncrosslinked oligonucleotide). Representative gels depict experiments performed at least three times.

The enhanced activity of SXE on fork-structured DNA prompted us to extend our analysis to see if mini-SLX4 augmented XPF-ERCC1 activity at an ICL. We made a fork-structured DNA substrate that contained a single site-specific nitrogen mustard-like crosslink close to the ss/ds junction (Figures 5A and S3C) (Angelov et al., 2009; Guainazzi et al., 2010). This simplified substrate was designed to mimic a stalled replication fork. 5′-radiolabeling this substrate should result in labels on both strands of the fork, allowing us to trace multiple products simultaneously. Based on the cleavage products we observed for a noncrosslinked fork, it was possible to predict the mass of products from the ICL incision, thereby assessing whether the lesion was cut at either side (unhooked). A cut site 3′ of the crosslink (green arrow) should result in labeled product much greater than (≫) 35 nt in mass. If this product was in turn cut 5′ to the lesion (red arrow), two radiolabeled products should be generated: >35 (the unhooked strand) and ≤15 nt (the strand adjacent to the ICL).

To test this prediction, we labeled the 5′ termini of the substrate and compared the reaction products of an ICL with a nonICL fork (YF), labeled on the leading strand template (Figure 5B). The experiment revealed that an initial product formed, migrating ≫35 nt (the size of a single noncrosslinked substrate arm of the YF substrate is 35 nt), indeed suggesting this must be a partially incised, crosslinked product (Figure 5B, green arrow). To verify this, we instead radiolabeled the 3′ termini of ICL and compared its digestion with 3′-labeled YF (Figure S5). This reaction produced a complementary picture, confirming that initial cleavage occurred 1 nt within the duplex (green arrowheads; Figures S5B and S5C). We noticed that as the reaction with the 5′-labeled ICL substrate proceeded, the initial ≫35 nt product diminished, with the concomitant accumulation of a smaller >35 product (Figure 5B, red bracket) and 15 nt product for both ICL and YF substrates (Figure 5B, red arrowhead). We therefore wanted to confirm if these products were linked to crosslink “unhooking.”

To test this, we first reacted 5′-radiolabeled ICL with an excess of XE (25 nM, 60 min) to prepare an incised ICL intermediate (Figure 5C, green box, ≫35 nt; explained in detail in Figures S6A and S6B). We used XE alone for this preparatory experiment because the reaction proceeded too rapidly with SXE, making it difficult to isolate the intermediate product. The product was PAGE-purified to serve as a substrate (“incised ICL”) in a second reaction, comparing the two enzyme complexes. Significantly, we found that on this incised ICL substrate, SXE complex rapidly catalyzed a second incision step, yielding the same two final products (>35 nt [red bracket] and 15 nt [red arrowhead]) as the full ICL substrate (Figure 5C). This indicates that SXE catalysis at the second site is not dependent on the presence of the 3′ arm. Furthermore, these bands (15 and >35 nt) form at similar intensity, indicating the formation of the two products is linked. The rate of product formation by SXE is in stark contrast to XE alone, which yielded almost no discernable product under these conditions. Therefore, mini-SLX4 not only influences XPF-ERCC1 catalysis at the first site but also rapidly induces a second incision.

### SLX4 Augments ICL Unhooking by XPF-ERCC1

**Figure 6:**
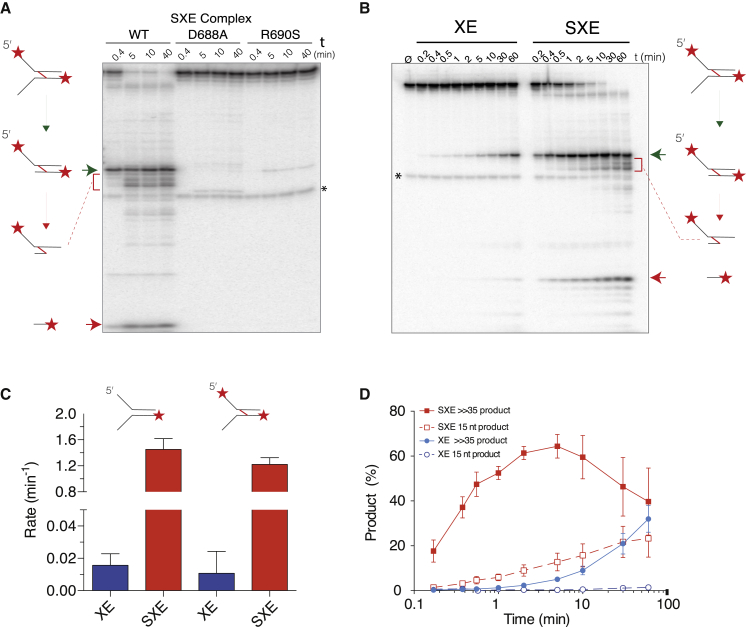
SLX4 Increases the Efficiency of XPF-ERCC1 ICL Unhooking (A) WT SXE or SXE harboring either XPF D678A or R690S mutations were incubated with the ICL substrate labeled at the 5′ end. ICL cleavage products are clearly seen with WT SXE, whereas SXE R690S (associated with human FA) shows very weak activity. The cleavage products are illustrated with a green arrow, red bracket, and red arrow (as described in Figure 5). A noncrosslinked oligonucleotide contaminant is marked with an asterisk. (B) Representative time course, comparing reaction of ICL substrate with either XE or SXE. (C) Rates of substrate turnover for ICL or equivalent noncrosslinked control, calculated from data presented in (B). (D) Graph representing the ICL product formation for XE (blue) and SXE (red) enzyme complexes. Filled symbols mark the first incision product (shown in B above as a green arrow), open symbols depict 15 nt product (B, red arrow). The accumulation of the 15 nt product is dependent on the first product and marks the “unhooking” of the crosslink. Assays were performed with 5 nM enzyme complex and ∼1.5 pM labeled substrate, incubated for the time indicated, quenched, and separated by 12% denaturing PAGE gel. Data in (C) and (D) are plotted from a minimum of three independent experiments; error bars represent SEM.

We confirmed nuclease activity on the ICL substrate was intrinsic to the SXE complex, by comparing WT SXE to that carrying XPF D688A. In addition, we tested SXE with XPF R690S point mutation (Figures 3A and S2), which results in a substitution close to the enzyme’s catalytic site and greatly diminishes nuclease activity (Figure 6A). R690S is equivalent to the mutation recently described in an FA patient (Bogliolo et al., 2013). This mutation in XPF almost abolished the activity of SXE on our crosslinked DNA substrate. These XPF mutants also had no activity in the absence of SLX4 (XE data not shown). Therefore, only the WT XPF protein in the SXE complex could efficiently catalyze ICL unhooking.

Having established that mini-SLX4 stimulated ICL unhooking by XPF-ERCC1, we wanted to quantify this effect in comparison with a noncrosslinked control (YF). XE and SXE shared the same cleavage sites on the YF and ICL substrate (Figure 5B). However, when we compared incision rates on the ICL substrate, the half-life for SXE was 34 s, compared to >60 min for XE (Figures 6A and 6B; Table S2). This is equivalent to a 110-fold increase in the catalytic rate of XPF-ERCC1 on a crosslinked substrate (Figure 6C). Reaction kinetics for the ICL were very similar to YF, indicating the crosslinked adduct does not pose a significant obstacle to SXE nuclease activity (Figure S4C). Furthermore, a time course confirmed the temporality of product formation from the ICL substrate (Figure 6D). The SXE reaction was initiated with the rapid accumulation of ≫35 nt product, peaking at 5 min, as this was subsequently converted to 15 nt product. For XE digestion, the complete unhooking reaction was limited by primary product formation (≫35 nt). Finally, we used our “incised ICL” to investigate the efficiency of the second incision by both nuclease complexes (Figures S6C and S6D). The time course revealed that XE was also markedly inefficient in making the second incision, compared to SXE (determined by 15 nt product formation). Taken together, these biochemical data suggest a critical function of SLX4 in ICL repair is in accelerating XPF-ERCC1 unhooking crosslinked DNA.

## Discussion

This work provides insight into the physiological and biochemical function of SLX4 in DNA repair. This important DNA repair protein is not merely a passive scaffold but rather acts as a factor that greatly stimulates the activity of the XPF-ERCC1 nuclease toward certain substrates. These features are exemplified by the ability of SLX4-XPF-ERCC1 complex to unhook an ICL at a synthetic replication fork.

Our previous genetic characterization of *Slx4*-deficient mice showed that homozygous animals exhibited a phenotype that shared many features with human FA. Consistent with this is the discovery that biallelic mutations in *SLX4* result in classical FA, making SLX4 the 15^th^ FA complementation group (FANCP) (Kim et al., 2011; Stoepker et al., 2011). Currently, the FANCP group consists of very few families, with individuals displaying a broad range of clinical features from varied developmental defects to bone marrow failure. Only one FANCP patient has developed malignant disease (a squamous cell carcinoma of the tongue) (Kim et al., 2011). Our observation that *Slx4*^*f3/f3*^ mice surviving to adulthood develop epithelial cancers establishes this DNA repair gene as a tumor suppressor.

A key feature of FA-deficient cells, including *Slx4* deficiency, is hypersensitivity to DNA interstrand crosslinking agents. This is due to an inability to repair a DNA crosslink. There is an emerging body of evidence showing that the upstream FA proteins are critical for orchestrating unhooking at the site of a crosslink (De Silva et al., 2000; Knipscheer et al., 2009), whereas downstream FA proteins are required for HR-mediated repair of the double-strand breaks generated by the incision step. SLX4 binds three nucleases, all of which are implicated in crosslink repair. Despite this, it has not been resolved at which stage of crosslink repair SLX4 and its associated nucleases act.

**Figure 7:**
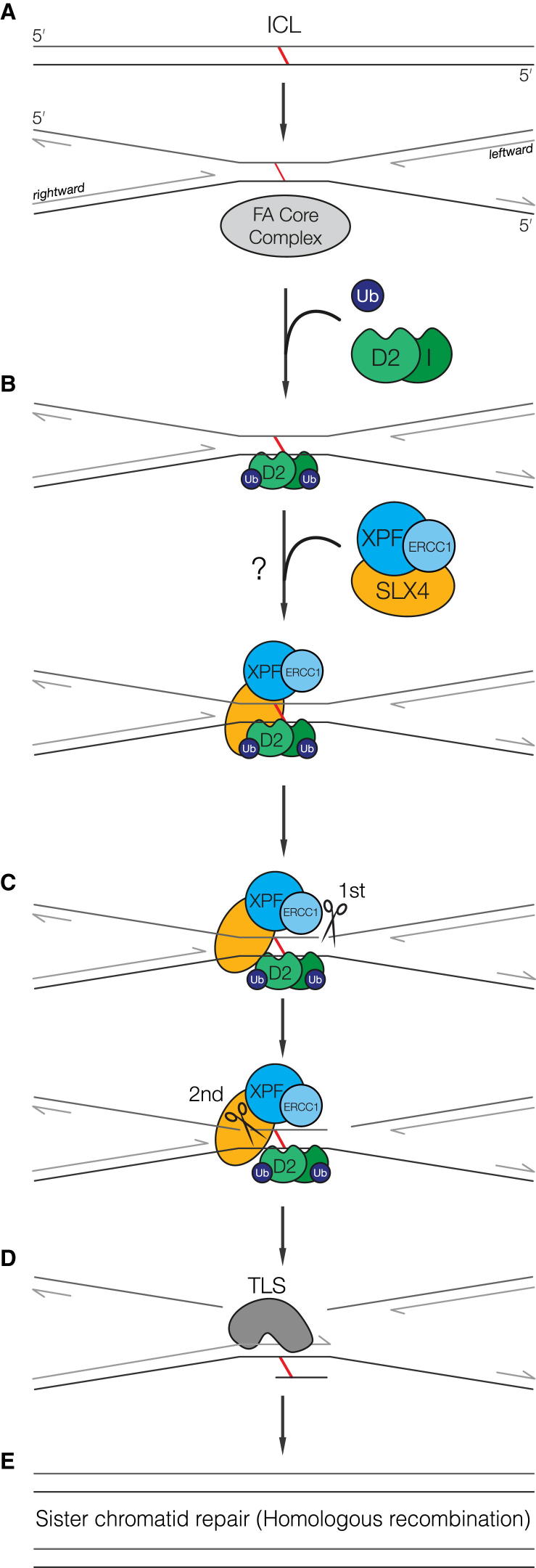
Model for the Role of SXE in ICL Repair (A) Monoubiquitylation of FANCD2 and FANCI (ID) by the FA core complex is required for interstrand crosslink recognition. (B) Ubiquitylated ID recruits SLX4 in complex with XPF-ERCC1 either directly or via an unidentified intermediary protein(s). SXE preference for a 3′ single-stranded arm suggests the molecular recognition of the crosslink is triggered by the convergence of both replication forks at the ICL. (C) The presence of the leftward fork would trigger SXE cutting first 3′ and possibly then 5′, unhooking the ICL. (D) The intact (adducted) parental strand could then serve as a template for the rightward fork extension by translesion synthesis. (E) The adducted base can then be removed by a combination of nucleotide excision repair and the newly synthesized chromatid used to repair the resulting DSB. SLX4 involvement in this process may additionally require the action of MUS81-EME1 and/or SLX1.

The work presented here shows that SLX4, in complex with XPF-ERCC1, is a far more potent nuclease than XPF-ERCC1 alone. Moreover, SLX4 imparts structural preference on XPF-ERCC1 toward DNA flaps and replication-like structures over stem-loop or bubble substrates (those bearing greater similarity to nucleotide excision repair substrates) without a free 3′ overhang. This stimulation of activity does not appear to be due to enhanced substrate binding, suggesting that its effect is more likely due to altered catalysis on specific substrates. Our data reveal that the individual proteins are present with 2:2:2 stoichiometry in the SXE complex, implying that each SXE complex contains two active sites of XPF. It is tempting to speculate that this may influence enzyme efficiency and potentially provides a mechanism by which enhanced catalysis is achieved. Our studies show that a minimal SXE complex is capable of dual incisions at either side of a DNA crosslink. The SXE complex primarily appears to recognize the 3′ arm of a fork and that cutting occurs 1 and 4 nt from the ss/ds junction. It is well known that XPF-ERCC1 is crucial for crosslink repair and that it can biochemically unhook a crosslink, but it is unclear the efficiency with which it achieves this (Fisher et al., 2008; Kuraoka et al., 2000). Our comparison of SLX4-XPF-ERCC1 complex and XPF-ERCC1 alone shows that SLX4 greatly stimulates this nuclease activity, which can be integrated into a model of ICL repair (Figure 7). Recently, it has been discovered that XPF-ERCC1 is critical for ICL incision in a *Xenopus* system, dependent upon monoubiquitylated FANCD2 (Klein Douwel et al., 2014; Knipscheer et al., 2009). Furthermore, it was previously shown that the leading strand template remains intact in this process (Räschle et al., 2008). While this may suggest a major difference between the activity we report and what is seen in *Xenopus*, a few points need to be taken into consideration. The *Xenopus* model of ICL repair involves two converging replication forks. It is therefore possible that the dual incisions we observe take their cue from the replication fork coming from the opposite direction toward the ICL. This would still leave an intact, adducted parental strand as template for TLS. The in vitro *Xenopus* system might also have additional factors that specifically restrict the activity of SXE nuclease complex to favor a particular arm (for example, preloading Rad51 onto ssDNA; Long et al., 2011). Indeed, although dependent on monoubiquitylated FANCD2, the mechanism by which SLX4-XPF-ERCC1 is recruited to site of the crosslink is uncertain. The recruitment may occur through the direct interaction of SLX4 and monoubiquitylated FANCD2 or indirectly, through an intermediary (Yamamoto et al., 2011).

Mini-SLX4 that interacts with only one nuclease XPF-ERCC1 does not fully rescue ICL sensitivity in SLX4-deficient cells, unlike the full-length protein. Therefore, parts of SLX4 distal to our truncation also contribute to ICL repair. The most likely candidates are the two other nucleases (MUS81-EME1 or SLX1), because cells deficient in them are sensitive to ICLs (Dendouga et al., 2005; Kratz et al., 2010; MacKay et al., 2010; McPherson et al., 2004). It is likely that either of these nucleases and/or just the distal part of SLX4 might play a role in later stages of ICL repair, such as in HR-mediated double-strand break repair. In summary, the experiments presented in this paper elucidate a genetic and biochemical function for murine SLX4, defining how this protein functions in ICL repair when bound only to XPF-ERCC1.

## Experimental Procedures

Please refer to the Supplemental Experimental Procedures for detailed methodology on strains, clonogenic assays, fluorescence-activated cell sorting analysis, cloning and mutagenesis (Table S4), protein expression and purification, size-exclusion chromatography-multiangle static light-scattering, mass spectrometry and nuclease substrates (including ICL synthesis), and comprehensive nuclease and binding assay conditions.

### Mice

*Btbd12*^*f3/f3*^ generated in the C57BL/6NTac background were described previously (Crossan et al., 2011). All animal experiments undertaken in this study were done so with the approval of the UK Home Office.

### Flow Cytometry

Flow cytometry was performed on bone marrow cells that were isolated from the femora and tibiae of mutant mice as described previously (Garaycoechea et al., 2012).

### Immunoprecipitation and Western Blot Analysis

Immunoprecipitation and western blot analysis were performed using the following antibodies: HA (Covance, MMS-101R), ERCC1 (Santa Cruz Biotechnology, FL297), XPF (Abcam, ab73720), anti-SLX4 (affinity purified rabbit serum immunized with SLX4 1-758), swine anti-rabbit (DEKO, P0399), and rabbit anti-mouse (DEKO, P0260).

### Nuclease Assays

All reactions were carried out in nuclease buffer (NB): 10–50 mM Tris (pH 8.0), 50 mM NaCl, 2 mM MgCl_2_, 1 mM TCEP, 0%–5% glycerol, and 0.1 mg/ml BSA (NEB) at 22°C. Reactions were analyzed on 12% denaturing PAGE gel, and data were fitted using GraphPad Prism. DNA is shown schematically in Figure S3, and sequences are listed in Table S3. Enzyme concentrations were calculated assuming the complexes were monomeric (i.e., in comparison the assays contain the same amount of XPF-ERCC1).

### Fluorescent Anisotropy Binding Assay

Synthetic oligonucleotides stem loop (FAM) and Y-shaped DNA fork (Cy5) were labeled with fluorescent probes on 5′ terminus as shown in Figure 3D. Enzyme complexes were prepared in 2-fold serial dilution, mixed 1:1 with DNA substrate (50 nM), and analyzed using PHERAstar (BMG). Enzyme concentrations were calculated assuming complexes were monomeric.

## Author Contributions

The study was conceived by K.J.P., M.R.G.H., J.S., and G.P.C. The manuscript was written by K.J.P., M.R.G.H., G.P.C., and J.S. All experiments were planned and executed by M.R.G.H., J.S., and G.P.C. J.I.G. generated cell lines and O.D.S. and S.M. synthesized the ICL substrate. SEC-MALS was performed by C.M.J.

## Supplemental Information

Document S1. Figures S1–S6, Tables S1–S4, and Supplemental Experimental Procedures

Document S2. Article plus Supplemental Information

## References

[bib1] Al-Minawi A.Z., Saleh-Gohari N., Helleday T. (2008). The ERCC1/XPF endonuclease is required for efficient single-strand annealing and gene conversion in mammalian cells. Nucleic Acids Res..

[bib2] Andersen S.L., Bergstralh D.T., Kohl K.P., LaRocque J.R., Moore C.B., Sekelsky J. (2009). Drosophila MUS312 and the vertebrate ortholog BTBD12 interact with DNA structure-specific endonucleases in DNA repair and recombination. Mol. Cell.

[bib3] Angelov T., Guainazzi A., Schärer O.D. (2009). Generation of DNA interstrand cross-links by post-synthetic reductive amination. Org. Lett..

[bib4] Auerbach A.D., Wolman S.R. (1976). Susceptibility of Fanconi’s anaemia fibroblasts to chromosome damage by carcinogens. Nature.

[bib5] Barnes D.E., Lindahl T. (2004). Repair and genetic consequences of endogenous DNA base damage in mammalian cells. Annu. Rev. Genet..

[bib6] Bhagwat N., Olsen A.L., Wang A.T., Hanada K., Stuckert P., Kanaar R., D’Andrea A., Niedernhofer L.J., McHugh P.J. (2009). XPF-ERCC1 participates in the Fanconi anemia pathway of cross-link repair. Mol. Cell. Biol..

[bib7] Bogliolo M., Schuster B., Stoepker C., Derkunt B., Su Y., Raams A., Trujillo J.P., Minguillón J., Ramírez M.J., Pujol R. (2013). Mutations in ERCC4, encoding the DNA-repair endonuclease XPF, cause Fanconi anemia. Am. J. Hum. Genet..

[bib8] Bowles M., Lally J., Fadden A.J., Mouilleron S., Hammonds T., McDonald N.Q. (2012). Fluorescence-based incision assay for human XPF-ERCC1 activity identifies important elements of DNA junction recognition. Nucleic Acids Res..

[bib9] Castor D., Nair N., Declais A.C., Lachaud C., Toth R., Macartney T.J., Lilley D.M., Arthur J.S., Rouse J. (2013). Cooperative control of Holliday junction resolution and DNA repair by the SLX1 and MUS81-EME1 nucleases. Mol Cell.

[bib10] Ceccaldi R., Parmar K., Mouly E., Delord M., Kim J.M., Regairaz M., Pla M., Vasquez N., Zhang Q.S., Pondarre C. (2012). Bone marrow failure in Fanconi anemia is triggered by an exacerbated p53/p21 DNA damage response that impairs hematopoietic stem and progenitor cells. Cell Stem Cell.

[bib11] Crossan G.P., van der Weyden L., Rosado I.V., Langevin F., Gaillard P.H., McIntyre R.E., Gallagher F., Kettunen M.I., Lewis D.Y., Brindle K., Sanger Mouse Genetics Project (2011). Disruption of mouse Slx4, a regulator of structure-specific nucleases, phenocopies Fanconi anemia. Nat. Genet..

[bib12] de Laat W.L., Appeldoorn E., Jaspers N.G., Hoeijmakers J.H. (1998). DNA structural elements required for ERCC1-XPF endonuclease activity. J. Biol. Chem..

[bib13] De Silva I.U., McHugh P.J., Clingen P.H., Hartley J.A. (2000). Defining the roles of nucleotide excision repair and recombination in the repair of DNA interstrand cross-links in mammalian cells. Mol. Cell. Biol..

[bib14] Demuth I., Digweed M., Concannon P. (2004). Human SNM1B is required for normal cellular response to both DNA interstrand crosslink-inducing agents and ionizing radiation. Oncogene.

[bib15] Dendouga N., Gao H., Moechars D., Janicot M., Vialard J., McGowan C.H. (2005). Disruption of murine Mus81 increases genomic instability and DNA damage sensitivity but does not promote tumorigenesis. Mol. Cell. Biol..

[bib16] Enzlin J.H., Schärer O.D. (2002). The active site of the DNA repair endonuclease XPF-ERCC1 forms a highly conserved nuclease motif. EMBO J..

[bib17] Evans E., Fellows J., Coffer A., Wood R.D. (1997). Open complex formation around a lesion during nucleotide excision repair provides a structure for cleavage by human XPG protein. EMBO J..

[bib18] Fekairi S., Scaglione S., Chahwan C., Taylor E.R., Tissier A., Coulon S., Dong M.Q., Ruse C., Yates J.R., Russell P. (2009). Human SLX4 is a Holliday junction resolvase subunit that binds multiple DNA repair/recombination endonucleases. Cell.

[bib19] Fisher L.A., Bessho M., Bessho T. (2008). Processing of a psoralen DNA interstrand cross-link by XPF-ERCC1 complex in vitro. J. Biol. Chem..

[bib20] Garaycoechea J.I., Patel K.J. (2014). Why does the bone marrow fail in Fanconi anemia?. Blood.

[bib21] Garaycoechea J.I., Crossan G.P., Langevin F., Daly M., Arends M.J., Patel K.J. (2012). Genotoxic consequences of endogenous aldehydes on mouse haematopoietic stem cell function. Nature.

[bib22] Garcia-Higuera I., Taniguchi T., Ganesan S., Meyn M.S., Timmers C., Hejna J., Grompe M., D’Andrea A.D. (2001). Interaction of the Fanconi anemia proteins and BRCA1 in a common pathway. Mol. Cell.

[bib23] Guainazzi A., Campbell A.J., Angelov T., Simmerling C., Schärer O.D. (2010). Synthesis and molecular modeling of a nitrogen mustard DNA interstrand crosslink. Chemistry.

[bib24] Hsia K.T., Millar M.R., King S., Selfridge J., Redhead N.J., Melton D.W., Saunders P.T. (2003). DNA repair gene Ercc1 is essential for normal spermatogenesis and oogenesis and for functional integrity of germ cell DNA in the mouse. Development.

[bib25] Kashiyama K., Nakazawa Y., Pilz D.T., Guo C., Shimada M., Sasaki K., Fawcett H., Wing J.F., Lewin S.O., Carr L. (2013). Malfunction of nuclease ERCC1-XPF results in diverse clinical manifestations and causes Cockayne syndrome, xeroderma pigmentosum, and Fanconi anemia. Am. J. Hum. Genet..

[bib26] Kim Y., Lach F.P., Desetty R., Hanenberg H., Auerbach A.D., Smogorzewska A. (2011). Mutations of the SLX4 gene in Fanconi anemia. Nat. Genet..

[bib27] Kim Y., Spitz G.S., Veturi U., Lach F.P., Auerbach A.D., Smogorzewska A. (2013). Regulation of multiple DNA repair pathways by the Fanconi anemia protein SLX4. Blood.

[bib28] Klein Douwel D., Boonen R.A.C.M., Long D.T., Szypowska A.A., Räschle M., Walter J.C., Knipscheer P. (2014). XPF-ERCC1 Acts in Unhooking DNA Interstrand Crosslinks in Cooperation with FANCD2 and FANCP/SLX4. Mol. Cell.

[bib29] Knipscheer P., Räschle M., Smogorzewska A., Enoiu M., Ho T.V., Schärer O.D., Elledge S.J., Walter J.C. (2009). The Fanconi anemia pathway promotes replication-dependent DNA interstrand cross-link repair. Science.

[bib30] Kratz K., Schöpf B., Kaden S., Sendoel A., Eberhard R., Lademann C., Cannavó E., Sartori A.A., Hengartner M.O., Jiricny J. (2010). Deficiency of FANCD2-associated nuclease KIAA1018/FAN1 sensitizes cells to interstrand crosslinking agents. Cell.

[bib31] Kuraoka I., Kobertz W.R., Ariza R.R., Biggerstaff M., Essigmann J.M., Wood R.D. (2000). Repair of an interstrand DNA cross-link initiated by ERCC1-XPF repair/recombination nuclease. J. Biol. Chem..

[bib32] Lindahl T. (1993). Instability and decay of the primary structure of DNA. Nature.

[bib33] Liu T., Ghosal G., Yuan J., Chen J., Huang J. (2010). FAN1 acts with FANCI-FANCD2 to promote DNA interstrand cross-link repair. Science.

[bib34] Long D.T., Räschle M., Joukov V., Walter J.C. (2011). Mechanism of RAD51-dependent DNA interstrand cross-link repair. Science.

[bib35] MacKay C., Déclais A.C., Lundin C., Agostinho A., Deans A.J., MacArtney T.J., Hofmann K., Gartner A., West S.C., Helleday T. (2010). Identification of KIAA1018/FAN1, a DNA repair nuclease recruited to DNA damage by monoubiquitinated FANCD2. Cell.

[bib36] McPherson J.P., Lemmers B., Chahwan R., Pamidi A., Migon E., Matysiak-Zablocki E., Moynahan M.E., Essers J., Hanada K., Poonepalli A. (2004). Involvement of mammalian Mus81 in genome integrity and tumor suppression. Science.

[bib37] McWhir J., Selfridge J., Harrison D.J., Squires S., Melton D.W. (1993). Mice with DNA repair gene (ERCC-1) deficiency have elevated levels of p53, liver nuclear abnormalities and die before weaning. Nat. Genet..

[bib38] Muñoz I.M., Hain K., Déclais A.C., Gardiner M., Toh G.W., Sanchez-Pulido L., Heuckmann J.M., Toth R., Macartney T., Eppink B. (2009). Coordination of structure-specific nucleases by human SLX4/BTBD12 is required for DNA repair. Mol. Cell.

[bib39] Niedernhofer L.J., Essers J., Weeda G., Beverloo B., de Wit J., Muijtjens M., Odijk H., Hoeijmakers J.H., Kanaar R. (2001). The structure-specific endonuclease Ercc1-Xpf is required for targeted gene replacement in embryonic stem cells. EMBO J..

[bib40] Niedzwiedz W., Mosedale G., Johnson M., Ong C.Y., Pace P., Patel K.J. (2004). The Fanconi anaemia gene FANCC promotes homologous recombination and error-prone DNA repair. Mol. Cell.

[bib41] Prasher J.M., Lalai A.S., Heijmans-Antonissen C., Ploemacher R.E., Hoeijmakers J.H., Touw I.P., Niedernhofer L.J. (2005). Reduced hematopoietic reserves in DNA interstrand crosslink repair-deficient Ercc1-/- mice. EMBO J..

[bib42] Räschle M., Knipscheer P., Enoiu M., Angelov T., Sun J., Griffith J.D., Ellenberger T.E., Schärer O.D., Walter J.C. (2008). Mechanism of replication-coupled DNA interstrand crosslink repair. Cell.

[bib43] Sengerová B., Allerston C.K., Abu M., Lee S.Y., Hartley J., Kiakos K., Schofield C.J., Hartley J.A., Gileadi O., McHugh P.J. (2012). Characterization of the human SNM1A and SNM1B/Apollo DNA repair exonucleases. J. Biol. Chem..

[bib44] Sijbers A.M., de Laat W.L., Ariza R.R., Biggerstaff M., Wei Y.F., Moggs J.G., Carter K.C., Shell B.K., Evans E., de Jong M.C. (1996). Xeroderma pigmentosum group F caused by a defect in a structure-specific DNA repair endonuclease. Cell.

[bib45] Simpson L.J., Sale J.E. (2003). Rev1 is essential for DNA damage tolerance and non-templated immunoglobulin gene mutation in a vertebrate cell line. EMBO J..

[bib46] Smogorzewska A., Matsuoka S., Vinciguerra P., McDonald E.R., Hurov K.E., Luo J., Ballif B.A., Gygi S.P., Hofmann K., D’Andrea A.D., Elledge S.J. (2007). Identification of the FANCI protein, a monoubiquitinated FANCD2 paralog required for DNA repair. Cell.

[bib47] Smogorzewska A., Desetty R., Saito T.T., Schlabach M., Lach F.P., Sowa M.E., Clark A.B., Kunkel T.A., Harper J.W., Colaiácovo M.P., Elledge S.J. (2010). A genetic screen identifies FAN1, a Fanconi anemia-associated nuclease necessary for DNA interstrand crosslink repair. Mol. Cell.

[bib48] Stoepker C., Hain K., Schuster B., Hilhorst-Hofstee Y., Rooimans M.A., Steltenpool J., Oostra A.B., Eirich K., Korthof E.T., Nieuwint A.W. (2011). SLX4, a coordinator of structure-specific endonucleases, is mutated in a new Fanconi anemia subtype. Nat. Genet..

[bib49] Su Y., Orelli B., Madireddy A., Niedernhofer L.J., Schärer O.D. (2012). Multiple DNA binding domains mediate the function of the ERCC1-XPF protein in nucleotide excision repair. J. Biol. Chem..

[bib50] Svendsen J.M., Smogorzewska A., Sowa M.E., O’Connell B.C., Gygi S.P., Elledge S.J., Harper J.W. (2009). Mammalian BTBD12/SLX4 assembles a Holliday junction resolvase and is required for DNA repair. Cell.

[bib51] Trujillo J.P., Mina L.B., Pujol R., Bogliolo M., Andrieux J., Holder M., Schuster B., Schindler D., Surrallés J. (2012). On the role of FAN1 in Fanconi anemia. Blood.

[bib52] Wang A.T., Sengerová B., Cattell E., Inagawa T., Hartley J.M., Kiakos K., Burgess-Brown N.A., Swift L.P., Enzlin J.H., Schofield C.J. (2011). Human SNM1A and XPF-ERCC1 collaborate to initiate DNA interstrand cross-link repair. Genes Dev..

[bib53] Wyatt H.D., Sarbajna S., Matos J., West S.C. (2013). Coordinated actions of SLX1-SLX4 and MUS81-EME1 for Holliday junction resolution in human cells. Mol. Cell.

[bib54] Yamamoto K.N., Kobayashi S., Tsuda M., Kurumizaka H., Takata M., Kono K., Jiricny J., Takeda S., Hirota K. (2011). Involvement of SLX4 in interstrand cross-link repair is regulated by the Fanconi anemia pathway. Proc. Natl. Acad. Sci. USA.

[bib55] Yoshikiyo K., Kratz K., Hirota K., Nishihara K., Takata M., Kurumizaka H., Horimoto S., Takeda S., Jiricny J. (2010). KIAA1018/FAN1 nuclease protects cells against genomic instability induced by interstrand cross-linking agents. Proc. Natl. Acad. Sci. USA.

[bib56] Zhou W., Otto E.A., Cluckey A., Airik R., Hurd T.W., Chaki M., Diaz K., Lach F.P., Bennett G.R., Gee H.Y. (2012). FAN1 mutations cause karyomegalic interstitial nephritis, linking chronic kidney failure to defective DNA damage repair. Nat. Genet..

