# Supplementary material for: Mouse SLX4 Is a Tumor Suppressor that Stimulates the Activity of the Nuclease XPF-ERCC1 in DNA Crosslink Repair
Source: Mol Cell. 2014 May 8;54(3):472–84. doi: 10.1016/j.molcel.2014.03.014 (PMC4017094; doi:10.1016/j.molcel.2014.03.014)
Supplement: Document S1. Figures S1–S6, Tables S1–S4, and Supplemental Experimental Procedures [file mmc1.pdf]

Molecular Cell, Volume *54*

Supplemental Information

Mouse SLX4 Is a Tumor Suppressor that Stimulates the Activity of the Nuclease XPF-ERCC1 in DNA Crosslink Repair

Michael R.G. Hodskinson, Jan Silhan, Gerry P. Crossan, Juan I. Garaycoechea,
Shivam Mukherjee, Christopher M. Johnson, Orlando D. Schärer, and Ketan J. Patel

Supplemental Data

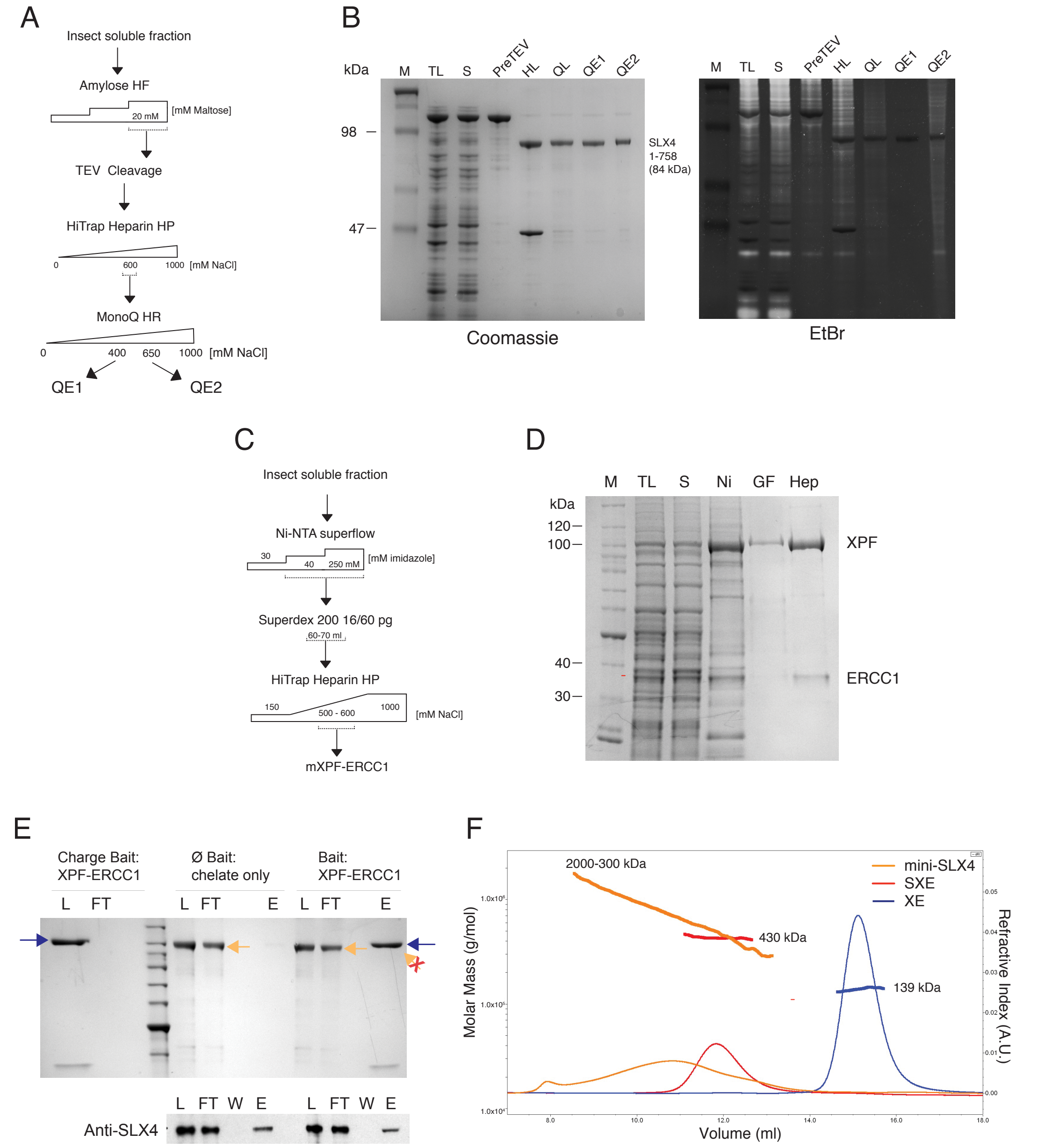

G

Identified Proteins (3/6)	Accession No	MW	% coverage SXE	XE
DNA repair endonuclease XPF	XPF_MOUSE	104 kDa	24%	39%
Structure-specific endonuclease subunit SLX4	SLX4_MOUSE	172 kDa	7.30%	0%
DNA excision repair protein ERCC-1	ERCC1_MOUSE	33 kDa	17%	16%
Structure-specific endonuclease subunit SLX4 1-758	n/a	84 kDa	15%	0%

Peptide Thresholds: 95.0% minimum
Protein Thresholds: 95.0% minimum and 5 peptides minimum
Peptide FDR: 1.8% (Prophet)
Protein FDR: 0.0% (Prophet)

Figure S1: Purification of individual components of SXE complex from insect Sf9 cells and analysis of their protein interactions.

(A): Diagram of purification of mini-SLX4. **(B):** Representative PAGE gel of mini-SLX4 purification, stained with Coomassie and with ethidium bromide (bright bands show presence of co-purifying DNA). M - marker, TL - total lysate, S – soluble fraction, PreTEV – MBP eluate, HL – Heparin Load, QL - monoQ Load, QE – Q

eluate in absence (1) or presence (2) of DNA **(C):** Purification Scheme for XE **(D):** XE purification analysed by SDS-PAGE, stained with Coomassie. M, TL, S (as above), Ni –Ni-chelate eluate, GF – gel filtration eluate, Hep – pooled heparin eluate. **(E):** Recombinant XPF-ERCC1 (His_6-tagged ERCC1) and mini-SLX4 do not associate when mixed *in vitro*. Top panel shows Coomassie stained gel of pull down assay with Recombinant XPF-ERCC1 (bait) and SLX4 (prey). XPF-ERCC1 was prebound to chelate resin (Charge Bait: XPF-ERCC1, L), absent in flow through (FT). SLX4 did not bind and appeared in FT of ø and bait resin (lower yellow arrow). Eluate, E - only bait proteins were released (blue arrow - XPF). Bottom Panel, WB detection of SLX4; its binding is non-specific. **(F):** MALS-SEC chromatograms of the refractive index signal. Evaluated masses are indicated by the thick horizontal lines (related to Figure 2E). SXE and XE were monodisperse with masses identical for all statistical averaging moments. The mass across the broad elution peak of mini-SLX4 varied between 800 – 2000 kDa, indicative of a polydisperse sample through self-association of the protein. This provides a possible explanation for its inability to form a complex with XE in vitro. Data are consistent with XE forming a heterodimer and SXE forming dimer of trimers at equimolar ratio (2:2:2). Predicted masses: mini-SLX4 - 84 kDa, XE - 135 kDa, SXE - 219 kDa. **(G):** Mass Spectrometry and peptide fingerprinting of purified XE and SXE, showing sequence coverage of isolated peptides. Notably, identified peptides for SXE include all protein components, whereas in XE, SLX4 peptides are undetectable.

Relates to **Figure 2**

A

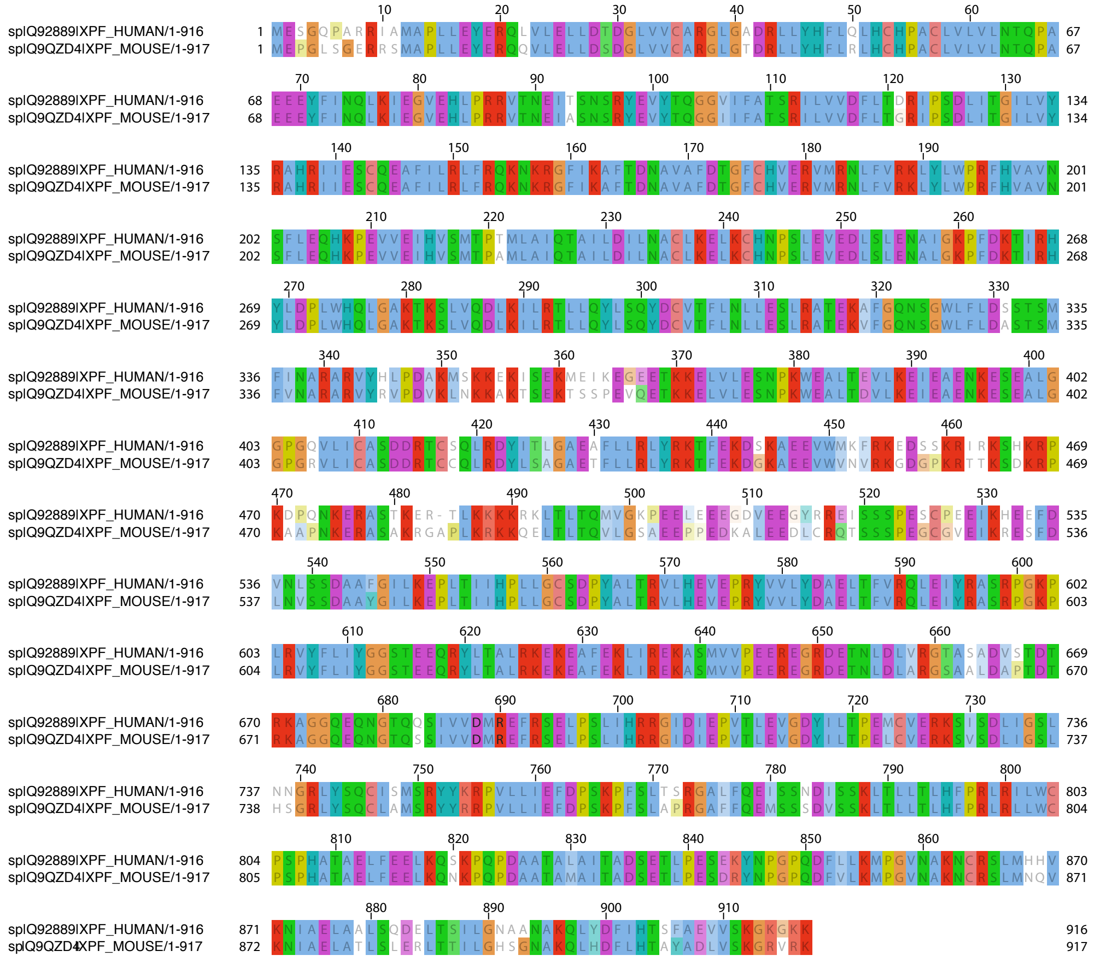

B

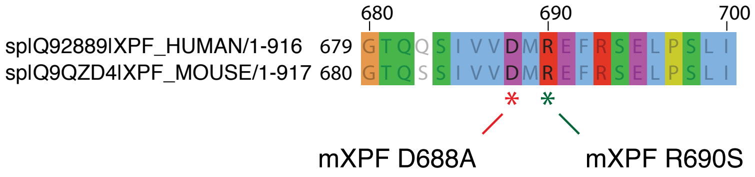

Figure S2: Alignment of mouse and human XPF indicating sites of mutated residues

(A): Human and mouse *XPF* orthologues were aligned using T-coffee. **(B):** Region of point mutants in proximity of catalytic site. Mouse D688A mutation responsible for coordination of catalytic metal is analogous mutation to human D687A (Enzlin and Scharer, 2002). R690S is an equivalent to that observed in FA patients (FA104, h*XPF* R689S) (Bogliolo et al., 2013). Positional numbering above the sequence is equivalent to the mouse protein.

Relates to **Figure 3** and **Figure 6**

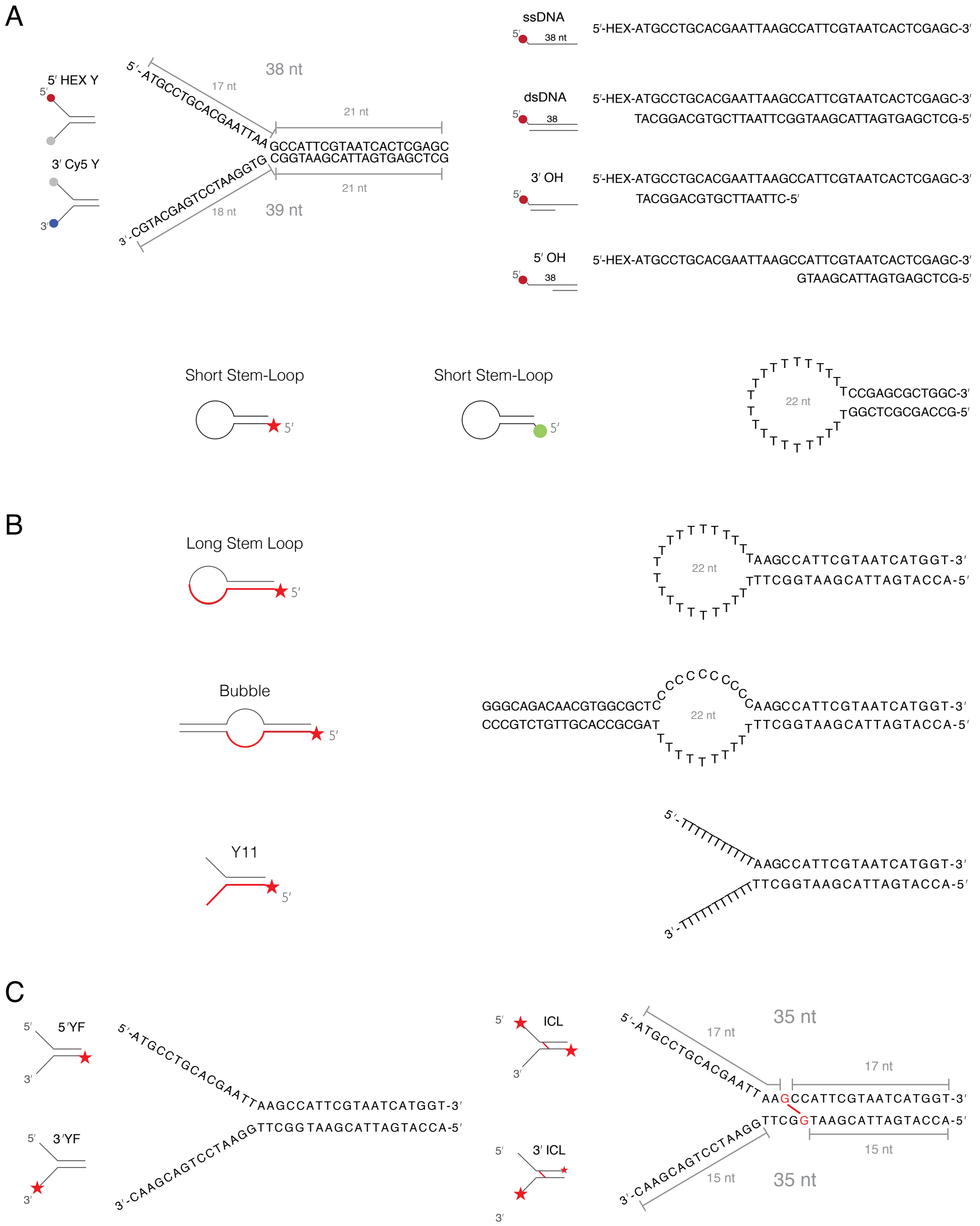

Figure S3: **Schematics of DNA substrates used in this study**

(A): Fluorescently labelled substrates, used in **Figure 3**: red dot – HEX; blue dot - Cy5; green circle – FAM (the same substrate, radiolabelled is marked with red star was used for kinetics in **Figure S4**). **(B):** Long stem-loop (stem-loop), Bubble Substrates and Y11 fork, used in **Figure 4** **(C):** Radiolabelled ICL substrate and non-crosslinked control, labelled either on the 5′ or 3′ end, used in **Figures 5 and 6**.

Relates to **Figures 3 to 6**

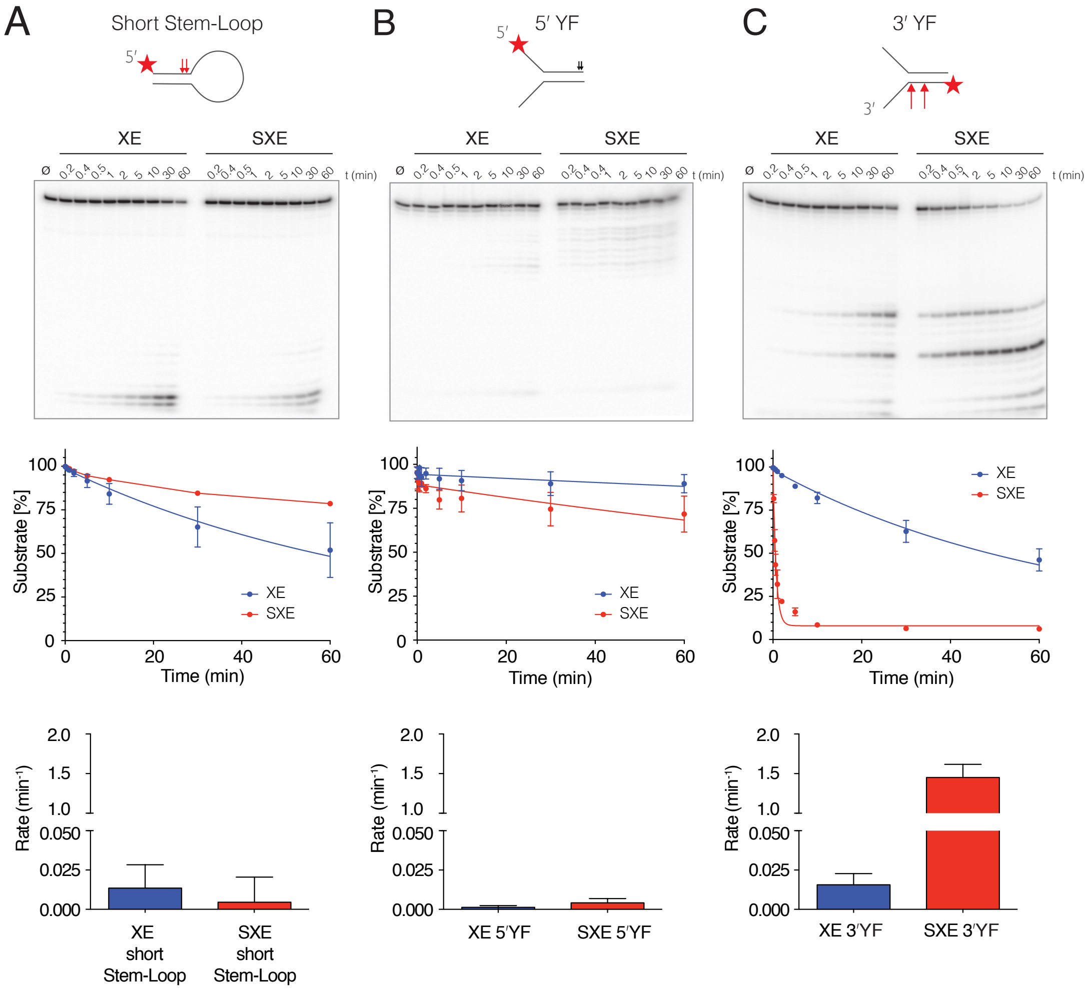

Figure S4: Kinetics of short stem-loop and DNA Y fork structure (YF) labelled on either strand

All substrates were radiolabelled at the 5' end **(A)**: SXE and XE were tested in their abilities to cleave a short stem-loop over a time course. Both XE and SXE cleaved this substrate, making two incisions 5′ of the loop (the canonical cleavage pattern of XPF-ERCC1) (de Laat et al., 1998). Note the different kinetics compared to the long stem-loop in described in Figure 4 **(B)**: Kinetics on the free 5′ strand of a Y structure. On the 5′ strand of YF structure, both complexes exhibited no clear or very weak activity **(C)**: Kinetics on the free 3′ strand of a Y structure. Both complexes formed two equivalent cleavage products – one corresponding to incision 1 nucleotide after the ss/dsDNA junction (19mer product) and a second cut within the duplex (15mer product). On measuring the catalytic rate of this reaction, however, we observed a difference between the enzyme complexes. SXE processed the 3′ arm of a YF substrate with a substrate half-life of 29 s, in contrast to 44 min for the equivalent XE-mediated catalysis. This corresponds to >90-fold increase in rate. Thus, mini-SLX4 strongly induces XPF-ERCC1 incision in a structure-specific manner on Y structured DNA. The decay of the substrate band was quantified and expressed as a

percentage of initial substrate – the red line corresponds to substrate with SXE and the blue for XE. In assays, 5 nM enzyme complex was mixed with ~1.5 pM [^{32}P] labelled substrate and incubated for time indicated, quenched and separated by 12% denaturing PAGE. Data were fitted from a minimum of three independent experiments using single exponential with Prism. Error bars represent SEM.

Relates to **Figure 3, 4 and 6**

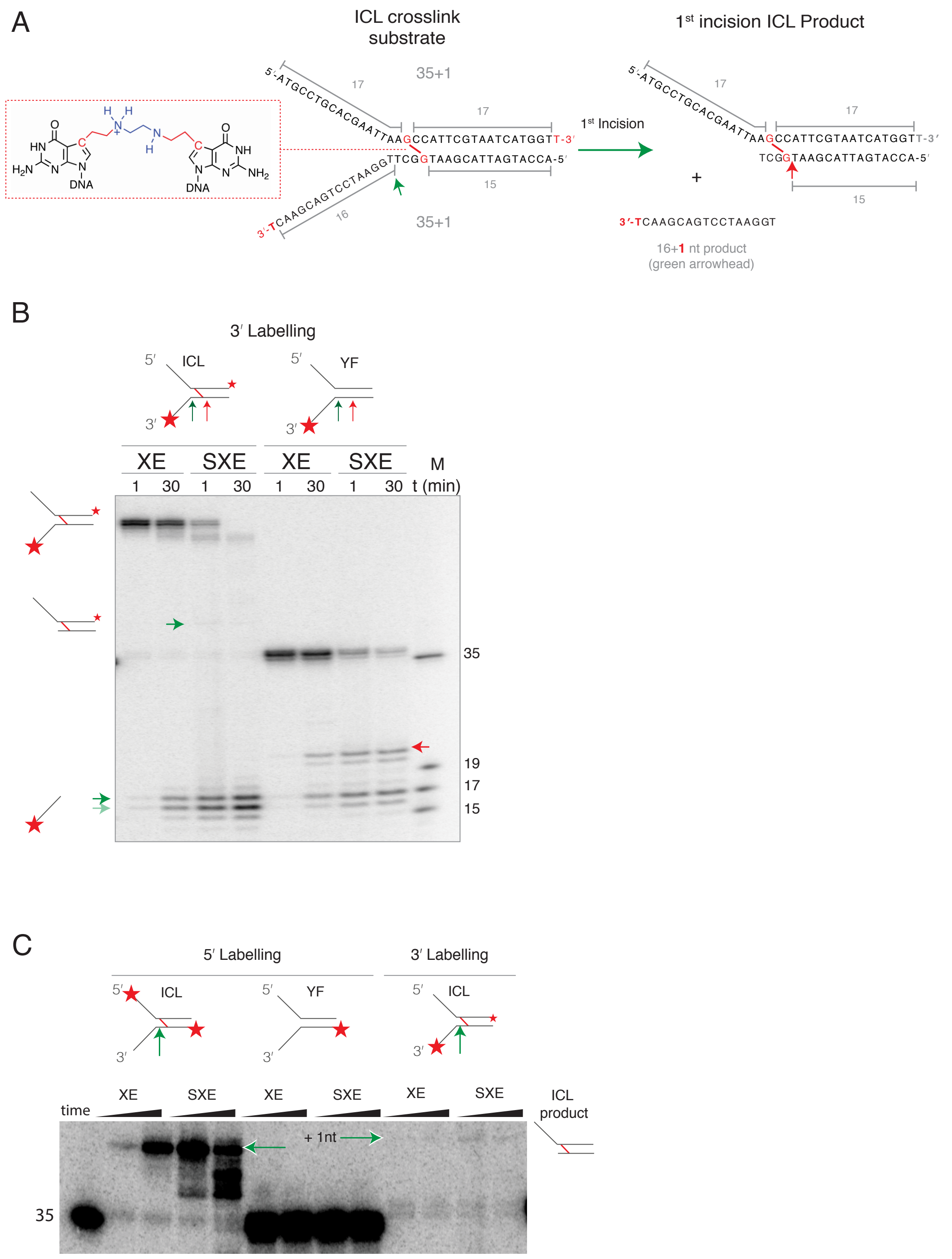

Figure S5: **Confirmation of reaction products using 3′-end labelled ICL and YF substrates**

(A): The ICL substrate, labelled by terminal transferase results in an additional nucleotide on the free 3′ arm and very weak labelling efficiency at the 3′ duplex (terminal transferase labels duplex DNA poorly (Bollum, 1974)). Products from this substrate therefore migrate 1 nt greater than the equivalent 5′-labelled products. **(B):**

The ICL substrate or an identical but non-crosslinked control (YF) was radiolabelled at the 3′ end and reacted with XE or SXE enzyme complexes and analysed by denaturing PAGE. Cleavage sites and reaction products corresponding to those illustrated in panel **A** are marked with green arrows (16 + 1 nt). Dual bands in both product and substrate have originated from 3′ labelling. **(C):** Comparison of 5′ and 3′ end labelling products from ICL or YF, illustrating the >>35 nt product resulting from the first incision. The image has been overexposed to reveal the faint >>35+1 crosslinked product which results from 3′-end labelled ICL digestion. The band is faint, primarily due to poor labelling (described above), combined with the weak activity we observe for SXE at the 3′ end of a duplex. All reactions contained 5 nM enzyme complex and ~1.5 pM substrate.

Relates to **Figure 5**

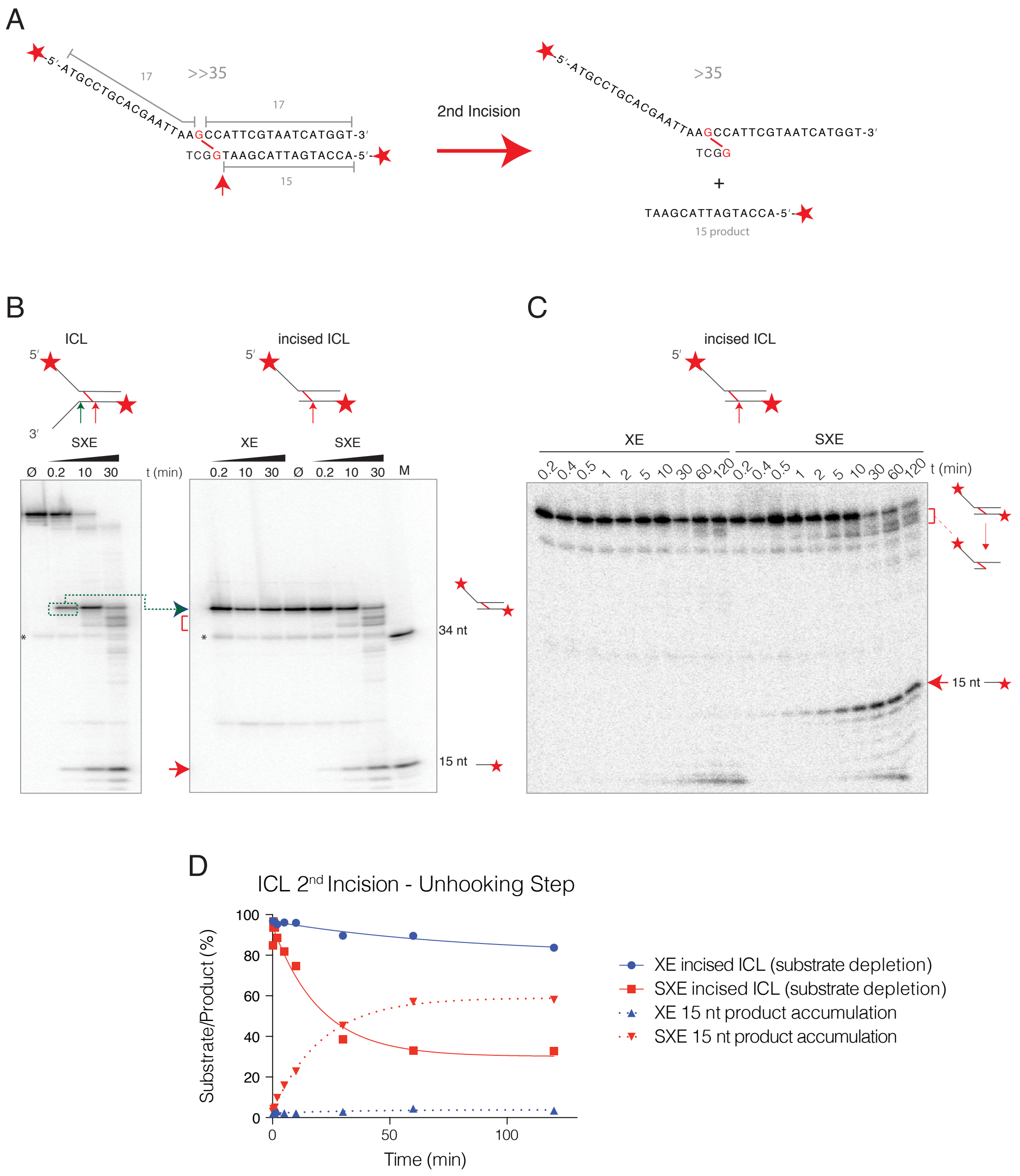

Figure S6: Kinetics of 2nd crosslink incision (unhooking)

(A): Scheme of second step in the unhooking reaction. The product (>>35 nt) of the first incision forms substrate for the second. The predicted mass of these products are >35 nt (the intact, adducted strand) and 15 nt. These are identical to the final products of the full ICL substrate, described in Figure 5A. **(B):** Left gel: ‘Incised ICL’ was generated from an excess of XE (20 nM for 1 h) and gel purified, as substrate for a second incision reaction. Data presented here are for illustrative purpose showing the same band (green box) after SXE digestion (5 nM for 12 s). Right gel: Reaction of enzyme complexes with the incised ICL substrate. Products from this reaction are identical to those generated from the full ICL substrate (>35 nt and 15 nt) - compare SXE, left and right gels. **(C):** Reaction kinetics of the second incision **(D):** Graphical

representation of the second incision comparing ‘incised ICL’ substrate depletion with concomitant accumulation of unhooked product (15 nt). Reactions were evaluated as described above for **Figures 4 and 6**. SXE efficiently catalysed the second incision, while XE processed this substrate slowly (XE 15 product accumulation was barely detectable after 2 h). All reactions in this figure contained 5 nM enzyme complex and ~1.5 pM substrate and were repeated three times.

Relates to **Figure 5** and **Figure 6**

Substrate	XE	SXE
	Rate ± SEM (min^{-1})	Rate ± SEM (min^{-1})
Long Stem Loop	0.023 ± 0.005	0.084 ± 0.003
Bubble	0.015 ± 0.008	0.078 ± 0.005
Y11	0.029 ± 0.0005	0.62 ± 0.004

Table S1: Reaction kinetics on substrates presented in Figure 4.

Substrate	XE	SXE
	Rate ± SEM (min^{-1})	Rate ± SEM (min^{-1})
Short Stem Loop	0.015 ± 0.002	0.0056 ± 0.0007
5′YF	0.0015 ± 0.0005	0.0048 ± 0.001
3′YF	0.014 ± 0.002	1.45 ± 0.12
ICL	0.01 ± 0.002	1.21 ± 0.12

Table S2: Rates comparing crosslinked ICL substrate and non-crosslinked substrate. Rates were determined as they were fitted to data displayed in **Figure 6** and **Figure S4**.

YA	5′-ATGCCTGCACGAATTAAGCCATTCGTAATCACTCGAGC-3′
YB	5′-GCTCGAGTGATTACGAATGGCGTGGAATCCTGAGCATGC-3′
3′ Overhang	5′-CTTAATTCGTGCAGGCAT-3′
5′ Overhang	5′-GCTCGAGTGATTACGAATG-3′
YA comp	5′-GCTCGAGTGATTACGAATGGCTTAATTCGTGCAGGCAT-3′
Short Stem Loop	5′-GCCAGCGCTCGG$(T)_{22}$CCGAGCGCTGGC-3′
Long Stem Loop	5′-ACCATGATTACGAATGGCTT$(T)_{22}$AAGCCATTCGTAATCATGGT-3′
Bubble A	5′-GGGCAGACAACGTGGCGCTCCCCCCCCCCCAAGCCATTCGTAATCATGGT-3′
Bubble B	5′-AAGCCATTCGTAATCATGGTTTTTTTTTTTTTTAGCGCCACGTTGTCTGCCC-3′
Y11A	5′-TTTTTTTTTTTAAGCCATTCGTAATCATGGT-3′
Y11B	5′-ACCATGATTACGAATGGCTTTTTTTTTTTTT-3′
YF(ICL)A	5′-ATGCCTGCACGAATTAAGCCATTCGTAATCATGGT-3′
YF(ICL)B	5′-ACCATGATTACGAATGGCTTGGAATCCTGACGAAC-3′

Table S3: DNA oligonucleotide sequences of the substrates used in this study. Annealed as shown in **Figure S3.**

Relates to Figures 3 – 6

**Flag-mSlx4 (1-758) primers for cloning into pExpress as a transition for pLox (BST) (SpeI sub-cloned)**	
mSlx4 758 pExpress Fw	5′-TTTGCTAGCATGGACTACAAAGATGACGATGACAAAGTTCCAGAGAGT GCTCCCAATGGCAAC-3′
mSlx4 758 pExpress Rw	5′-TTCTAGATATCAATCCTGGGCCTCTGCTTTCCCTGCC-3′
**mSLX4 primers introducing MBP-(TEV site)-mSLX4 1-758 cloning into pOPT vector**	
mSlx4 MBP pOPT Fw	5′-AAACATATGGTTCCAGAGAGTGCTCCC-3′
mSlx4 MBP pOPT Rw	5′-TTTACGCGTTCAATCCTGGGCCTCTGCTTTCCCT-3′
**MBP-Slx4 fusion protein primers sub-cloning for baculoviral production**	
mSlx4 MBP baculo Fw	5′-TTTGTCGACACCATGAAAATCGAAGAAGGTAAACT-3′
mSlx4 MBP baculo Rw	5′-AAAGCGGCCGCTCAATCCTGGGCCTCTGCTTTCCCTGCC-3′
**Cloning primers for XPF WT for baculoviral production**	
mXpf WT Fw	5′-TTTGTCGACACCATGGCGCCGCTGTTGGAGTA-3′
mXpf WT Rw	5′-AAAGCGGCCGCTCACTTTCTCACTCTGCCTTTGG-3′
**Cloning primers for ERCC1 WT for baculoviral production**	
mErcc1 HT FW	5′-TTTGTCGACACCATGGACCCTGGGAAGGACGAGG-3′
mErcc1 HT RW	5′- AAAGCGGCCGCTCAATGGTGATGATGGTGATGTCGAGGCACTTTGAGGAAGGGT TCG-3′
**Mutagenesis PCR primers to generate point mutants of XPF**	
mXpf D688A Fw	5′-GTGGCCATGCGTGAGTTTCGGAGCGAGCTCCCATCT-3′
mXpf D688A Rw	5′-CTCACGCATGGCCACCACGATGCTGGACTGGGTGCC-3′
mXpf D690S Fw	5′-GTGGACATGTCTGAGTTTCGGAGCGAGCTCCCATCT-3′
mXpf D690S Rw	5′-CTCAGACATGTCCACCACGATGCTGGACTGGGTGCC-3′

Table S4: DNA oligonucleotide sequences used for cloning and mutagenesis in this study

Related to **Experimental Procedures**

Supplemental Experimental Procedures

Mice. *Btbd12*$^{f3/f3}$ generated in the C57BL/6NTac background, were described previously (Crossan et al., 2011). All animals were maintained in specific pathogen-free conditions. In individual experiments, all mice were matched for age and gender. All animal experiments undertaken in this study were done so with the approval of the UK Home Office.

Histological analysis was performed on tissues that had been fixed in neutral buffered formalin for 24 h. The samples were then paraffin embedded and 4 µm sections were cut prior to staining with haematoxylin and eosin.

Irradiation of mice was performed using a Cs-137 GSR C1m blood irradiator (Gamma-Service Recycling Gmbh, Germany). Mice received a dose of 900 rads of total body irradiation, split between two equal doses, separated by 4 h. Mice received prophylactic enrofloxacin (Baytril, Bayer) in the drinking water for 7 d before irradiation.

CFU-S assays were performed as described previously (Garaycoechea et al., 2012). To assess the frequency of CFU-S in mutant mice, total bone marrow was flushed from the femora and tibiae of mutant mice and appropriate controls. Nucleated cells were enumerated using a solution of 3% acetic acid and methylene blue. The $1x10^5$ mutant bone marrow cells were then injected intravenously into recipient irradiated mice that had been lethally irradiated. After 10 d the spleens were fixed in Bouin's solution (Sigma), the number of colonies counted and made relative to the number of total bone marrow cells injected.

Flow cytometry was performed on bone marrow cells that were isolated from the femora and tibiae of mutant mice as described previously (Garaycoechea et al., 2012). The following antibodies were used to stain for HSCs: FITC-conjugated lineage cocktail with antibodies anti CD4 (clone H129.19, BD Pharmingen), CD3e (clone 145-2C11, eBioscience), Ly-6G/Gr-1 (clone RB6-8C5, eBioscience), CD11b/Mac-1 (clone M1/70, BD Pharmingen), CD45R/B220 (clone RA3-6B2, BD Pharmingen), Fcϵ R1α (clone MAR-1, eBioscience), CD8a (clone 53-6.7, BD Pharmingen), CD11c (clone N418, eBioscience) and TER-119 (clone Ter119, BD Pharmingen), anti c-Kit (PerCP-Cy5.5, clone 2B8, eBioscience), anti Sca-1 (PE-Cy7, clone D7, eBioscience).

Cloning and Mutagenesis primers used in this study are listed in **Table S4**. For complementation analysis the *Slx4* cDNA was amplified to encode an N-terminal Flag-Tag and subcloned into pExpress (as previously described (Crossan et al.,

2011). For recombinant protein expression mouse, *Slx4* cDNA was amplified and cloned into pOPTM (a generous gift from Dr Olga Perisic). The *malE-Slx4* gene fusion was then subcloned into pDEST8 (Life Technologies), as a SalI-NotI fragment. Mouse cDNA for *Ercc1* was amplified to encode a C-terminal 6xHis tag and *Xpf* without tags; similarly cloned into pDEST8 as SalI-NotI fragments. Site directed mutagenesis of *Xpf* was performed using In-Fusion cloning, according the manufacturer's instructions (Clontech). Bacmids and virus were prepared according to standard methods.

MEF survival assays were performed essentially as described previously (Crossan et al., 2011). Briefly, 1000 cells were seeded into each well of a flat bottom 96 well plate before being exposed to different doses of Mitomycin C (MMC). Cells were incubated with MMC for 4 d, before being pulsed with MTS reagent (Promega), after 2 h the wells were processed and the absorbance was measured at 492 nm – providing a marker for cell proliferation/viability.

Immunoprecipitation and Western Blot analysis was performed using the following antibodies: HA (Covance, MMS-101R), ERCC1 (Santa Cruz Biotechnology, FL297), XPF (Abcam, ab73720), anti-SLX4 (affinity purified rabbit serum immunised with SLX4 1-758), swine anti-rabbit (DEKO, P0399), rabbit anti mouse (DEKO, P0260). For immunoprecipitation, cells were lysed in 750 µl NENT buffer (50 mM Tris-HCl pH 7.5, 150 mM NaCl, and 0.5% NP-40 (Calbiochem)) supplemented with protease inhibitors (Roche Diagnostics). Whole cell extract (1 mg) was incubated for 6 h with 20 µl of anti-FLAG gel M2 (Sigma-Aldrich). After 4 washes in lysis buffer, the immunoprecipitates were eluted in Tris-glycine SDS sample buffer, analysed by 4-12% Tris-Acetate SDS-PAGE (Invitrogen) and the proteins detected by Western blotting.

Expression and Purification of protein complexes. Sf9 insect cells (2 L) were infected at 1-2 x 10^6 cell/ml with tertiary recombinant baculovirus, grown for 68 h and harvested. For XE and SXE complexes all purification steps were carried out in a variation of NENT buffer containing 20 mM Tris pH 8.0, 150-400 mM NaCl, 10% glycerol, 5 mM TCEP and protein inhibitors cocktail (see Figure 2D for schematics). Cells were homogenised in NENT buffer supplemented with 40 mM imidazole pH 8.0 and 0.1% NP-40 followed by nickel affinity chromatography on NTA agarose (QIAGEN) and proteins were eluted with NENT buffer, supplemented with 250 mM imidazole pH 8.0. For SXE complex an MBP affinity step (NEB, E8022L) was included and complex was eluted with 20 mM maltose. MBP tag was cleaved with TEV protease O/N at 4˚C. Complexes were diluted with NENT buffer to reduce salt to 200 mM NaCl and loaded on HP Heparin column (GE Healthcare) and eluted with

gradient of salt. Concentrated samples were purified on HiLoad Superose 6 (GE Healthcare) and combined fractions were flash frozen in liquid N_2. XPF point mutants in XE and SXE complexes were purified by an identical procedure to WT. Mini-SLX4 alone was purified, essentially as described above (omitting the chelate step) with the addition of a monoQ HR column step (eluted over a 0 - 1 M NaCl gradient).

Analytical size-exclusion chromatography (SEC). Mini-SLX4, XE and SXE complexes were buffer exchanged (20 mM Tris 8.0, 50 mM NaCl and 5% glycerol, 1 mM TCEP) and 50 µl of 5 µM samples were injected onto a PC3.2/30 (2.4 ml) Superose 6 gel filtration column (GE Healthcare). Fractions (50 µl) were collected and analysed by 4-12% SDS–PAGE, stained with Coomassie brilliant blue.

Light Scattering. SEC-MALS experiment was performed using a Wyatt Heleos II 18 angle light scattering instrument coupled to a Wyatt Optilab rEX online refractive index detector. Detector 12 was replaced with Wyatt's QELS detector. Samples for analysis were resolved on a Superose 6 10/300 analytical gel filtration column (GE Healthcare) running at 0.5 ml/min in 20 mM Tris pH 8.0, 150 mM NaCl, 5 mM TCEP buffer before passing through the light scattering and refractive index detectors in a standard SEC MALS format. Protein concentration was determined from the excess differential refractive index based on 0.186 ΔRI for 1 mg/ml. Concentration and the observed scattered intensity were used to calculate absolute molecular mass from the intercept of the Debye plot using Zimm's model as implemented in Wyatt's ASTRA software.

Mass spectrometry. Protein samples were reduced, alkylated and digested with trypsin, using the Janus liquid handling system (PerkinElmer, UK). The digests were subsequently analysed by LC-MS/MS on an Orbitrap Velos mass spectrometer (ThermoScientific, San Jose, USA). LC-MS/MS data were searched against a protein database (UniProt KB) using the Mascot search engine programme (Matrix Science, UK) (Perkins et al., 1999). MS/MS data were validated using the Scaffold programme (Proteome Software Inc., USA) (Keller et al., 2002). All data were additionally interrogated manually.

Fluorescent anisotropy binding assay. Synthetic oligonucleotides stem-loop (FAM) and Y-shaped DNA Fork (Cy5) were labelled with fluorescent probes on 5′ terminus as shown Figure 3D. Enzyme complexes were prepared in 2-fold serial dilution then mixed 1:1 with DNA substrate (50 nM) and analysed using PHERAstar (BMG). The assays were performed in 20 mM Tris pH 8.0, 50 mM NaCl, 5 % Glycerol, 1 mM TCEP, 0.1 mg/ml BSA. Data were fitted using predefined one-site specific binding equation in GraphPad Prism.

Nuclease assay standard reaction conditions. All reactions were carried out in nuclease buffer NB: 10-50 mM Tris 8.0, 50 mM NaCl, 2 mM $MgCl_2$, 1 mM TCEP, 0-5 % Glycerol, 0.1 mg/ml BSA (NEB) at 22˚C. Data were fitted using GraphPad Prism. Unless otherwise stated DNA substrate sequences for individual experiments are shown schematically in **Figure S3** and listed in **Table S3**. Cross-linked substrate was synthesised as described below.

Nuclease assay with fluorescently labelled substrate. DNA substrates (ssDNA, ds DNA, 3′ overhang and 5′ overhang) were 5′ labelled with HEX. Y structures were labelled on the free 5′ arm with HEX, and on the free 3′ arm with Cy5. The short stem-loop was labelled with FAM on the 5′ end. Substrates were purified on 15% denaturing PAGE gel, desalted and annealed by slow cooling from 90˚C. Reactions were initiated by generation of equimolar mixture [100 nM] of given substrate and enzyme XE, SXE and their XPF point mutant D688A. After 10 min reactions were quenched with 80% formamide, 200 mM NaCl, 10 mM EDTA, 0.01% bromophenol blue and analysed on 15% denaturing PAGE. The salt was present in quenching mixture to avoid protein denaturation on the DNA, which caused band smearing.

^{32}P Labelling and Oigonucleotide Annealling. DNA substrates (stem-loops, bubble DNA substrate, Y fork DNA structure and cross-linked fork substrates) were labelled on 5′ with γ-[32P] ATP using Optikinase (GE Healthcare). DNA labelling (10 pmol) at 3′ end with DNA terminal transferase (20 U) in 1 x TdT buffer, supplemented with 2.5 mM $CoCl_2$, α-[32P] CordycepinTP (50 μCi) in a 50 μl reaction at 37˚C, according to standard methods (NEB). Samples were desalted and the α-[32P] ATP or (or γ-[32P] ATP) incorporation was estimated by scintillation counting. Oligonucleotides were annealed by slow cooling from 95˚C and purified from 12% native PAGE gel.

DNA nuclease assay with ^{32}P labelled substrates. Typical reactions (80 μl) contained 80,000 cpm radiolabelled DNA substrate (~1.5 pM) and 5 nM XE or SXE in nuclease buffer, NB. Reactions were incubated at 22°C and quenched at the indicated time points with 80% formamide, 200 mM NaCl, 10 mM EDTA, 0.01% bromophenol blue. Reactions were analysed on 12% denaturing PAGE gel (1 x TBE, 7 M urea, 12% 19:1 acrylamide/bis-acrylamide 19:1). Dried gels were exposed for 12 - 15 h and scanned by Typhoon PhosphoImager (GE Healthcare). Band intensities were determined using *ImageQuant.* Relative substrate depletion was plotted against the time and fitted by single-exponential decays using GraphPad Prism. The rates of substrate depletion for stem-loop, both strands of Y-fork and crosslinked Y-fork, were plotted into the bar chart to underline the rate enhancement. All data are represented as mean ± SEM. The reaction rates representing the best fit to the data are listed in **Table S1.**

Synthesis of Y-shaped ICL substrate. Single stranded oligonucleotides Y1 (5′-ATGCCTGCACGAATTAAG*CCATTCGTAATCATGGT-3′) and Y2 (5′-CAAGCAGTCCTAAGGTTCGG*TAAGCATTAGTACCA-3′) containing the modified phosphoramidite G* (G* = 7-deaza-(2,3-diacetoxy-propyl)-2′-deoxyguanosine) were synthesized on Expedite 8909 DNA Synthesizer, deprotected using concentrated NH_3 and purified on a 15% denaturing PAGE gel. ICL formation was achieved using oxidation of the diol with $NAIO_4$ followed by double reducitve amination with N,N-dimethylethylenediamine and NBH_3CN using our established protocol (Angelov et al., 2009; Guainazzi et al., 2010).

Supplemental References

Bollum, F.J. (1974). Terminal deoxynucleotidyl transferase, Vol 10 (New York: Academic Press).

Keller, A., Nesvizhskii, A.I., Kolker, E., and Aebersold, R. (2002). Empirical statistical model to estimate the accuracy of peptide identifications made by MS/MS and database search. Anal Chem *74*, 5383-5392.

Perkins, D.N., Pappin, D.J.C., Creasy, D.M., and Cottrell, J.S. (1999). Probability-based protein identification by searching sequence databases using mass spectrometry data. Electrophoresis *20*, 3551-3567.
